# The risks of cancer development in systemic lupus erythematosus (SLE) patients: a systematic review and meta-analysis

**DOI:** 10.1186/s13075-018-1760-3

**Published:** 2018-12-06

**Authors:** Lebin Song, Yi Wang, Jiayi Zhang, Ninghong Song, Xiaoyun Xu, Yan Lu

**Affiliations:** 1 Department of Dermatology, The First Affiliated Hospital of Nanjing Medical University, No. 300 Guangzhou Road, Nanjing, 210029 China; 2 Department of Urology, The First Affiliated Hospital of Nanjing Medical University, No. 300 Guangzhou Road, Nanjing, 210029 China

**Keywords:** Systemic lupus erythematosus, Cancer, Meta-analysis

## Abstract

**Background:**

Although accumulating data have suggested the development of cancer in systemic lupus erythematosus (SLE) patients, these results remain inconsistent. To examine such a putative association, this analysis reports the association between SLE and the risks of 24 cancer types.

**Methods:**

Online databases PubMed, EMBASE, and Web of Science were searched comprehensively for eligible studies, published up to 15 May 2018. Pooled standardized incidence rates (SIRs) with 95% confidence intervals (CIs) were utilized to reveal their associations.

**Results:**

A total of 24 eligible studies were ultimately enrolled. Our results indicated that SLE was associated with increased risk of overall cancers, cancer risk in both genders, non-Hodgkin’s lymphoma, Hodgkin’s lymphoma, leukemia, multiple myeloma, cervix, vagina/vulva, renal, bladder, esophagus, gastric, hepatobiliary, lung, oropharynx, larynx, non-melanoma skin, and thyroid cancers. Additionally, SLE could reduce the risk of prostate cancer and cutaneous melanoma; however, it was not significantly associated with breast, uterus, ovarian, pancreatic, colorectal, or brain cancers.

**Conclusions:**

Our results shed light SLE being correlated with increased risk for 16 involved cancers and decreased risk for prostate cancer and cutaneous melanoma. This comprehensive meta-analysis provides epidemiological evidence supporting the associations between SLE and cancer risk. This evidence could be utilized to drive public policies and to help guide personalized medicine to better manage SLE and reduce associated cancer morbidity and mortality.

**Electronic supplementary material:**

The online version of this article (10.1186/s13075-018-1760-3) contains supplementary material, which is available to authorized users.

## Background

Systemic lupus erythematosus (SLE), defined as a complex and chronic inflammatory autoimmune disease, is characterized by the production of autoantibodies, complement activation, and immune complex deposition, which can be directed against almost any organ system in a heterogeneous array of clinical manifestations [1]. SLE predominantly occurs in young and middle-aged people with a female to male ratio of 10:1 [2], and the kidneys and the skin are the most intensively affected organs [3, 4]. Regarding the incidence and prevalence of SLE, the highest estimates of disease are in North America and in people of African ethnicity [5]. Major causes of morbidity and mortality in SLE patients include infection, cancer, renal failure, myocardial infarction, and central nervous system disease [6–9]. Due to early meticulous diagnosis and the progress of treatment, survival rates for SLE patients have increased remarkably in recent decades. Despite their increased life expectancy, these patients still have two- to five-times the risk of death compared with the general population, not only for all-cause mortality but also for mortality from cancer [10]. As a result, more attention should be paid to the risks of cancer development in patients with SLE.

Until now, a growing amount of research has attempted to reveal the incidence of cancers in SLE patients, and several studies have successfully demonstrated that SLE is significantly associated with increased risks of thyroid cancer [11], cervix cancer [12], and hematologic cancer [13]. With more than 25 years of follow-up, Tallbacka et al. confirmed that patients with SLE had an increased risk of cancer, particularly non-Hodgkin’s lymphoma and kidney cancer [14]. Moreover, Chen et al. reported a decreased risk of prostate cancer and bladder cancer in a cohort of 11,763 lupus patients in Taiwan [15]. There are also several studies suggesting that no direct associations exist between particular cancers and SLE. For instance, Rezaieyazdi et al. suggested that SLE was not dramatically related with the risk of breast cancer [16]. However, their results were not comprehensive, and some outcomes remained inconsistent. Hence, this meta-analysis was conducted to comprehensively shed light on the relationship between SLE and various cancers.

Here, 24 human malignant neoplasms were systematically divided into six systemic groups (lymphatic and hematopoietic cancers, reproductive cancers, urinary cancers, digestive cancers, respiratory cancers, and others) which were evaluated respectively. The outcomes from each could be utilized as a reference for future clinical management.

## Materials and methods

### Search strategy

To investigate the potential relationship between SLE and various cancers, relevant articles were comprehensively and systematically identified from the online databases PubMed, EMBASE, and Web of Science, published up to 15 May 2018. Pooled standardized incidence rates (SIRs) with 95% confidence intervals (CIs) were utilized to clarify their correlations. The search strategy mainly consisted of the following keywords in combination with Medical Subject Headings (MeSH) terms and text words: (“Systemic Lupus Erythematosus” or “Lupus Erythematosus Disseminatus” or “Libman-Sacks Disease” or “Libman Sacks Disease”) and (“Neoplasia” or “Neoplasias” or “Neoplasm” or “Tumors” or “Tumor” or “Cancer” or “Cancers” or “Malignant Neoplasms” or “Malignant Neoplasm” or “Malignancy” or “Malignancies”). The subsequent meta-analysis was strictly performed according to the Preferred Reporting Items for Systematic Reviews and Meta-Analyses (PRISMA) statement [17].

### Inclusion/exclusion criteria

Relevant articles finally enrolled in this meta-analysis met the following criteria: 1) language was restricted to English publications; 2) patients were diagnosed with SLE; 3) focused on the incidence of cancers in SLE patients; and 4) sufficient data provided by means of SIRs with 95% CIs. The major exclusion criteria were: 1) non-English articles; 2) duplicates or reviews or letters or case reports or comments or editorials; 3) simple description without comparison; 4) absence of key information; and 5) unrelated to SLE or cancers.

### Data extraction and quality assessment

The whole selection process and eligible articles were independently determined by two blinded reviewers (LS and YW) based on the inclusion and exclusion criteria. Disagreements were addressed by consultation with a third reviewer (JZ). The following information was extracted from enrolled articles: 1) first author’s name; 2) year of publication; 3) data origin; 4) calendar period; 5) number of patients (along with gender); 6) SLE diagnosis; 7) follow-up time (years); 8) the Newcastle-Ottawa Scale (NOS) scores; 9) observed/expected events; and 10) SIRs with 95% CIs. Methodological quality assessment of each eligible article was assessed with the NOS (http://www.ohri.ca/programs/clinical_epidemiology/oxford.htm), one of the most useful scales for evaluating the quality of nonrandomized studies [18]. The NOS scale utilizes a star rating system (with scores ranging from 0 to 9) to evaluate the quality of each study. Studies awarded six or more stars are regarded as high quality.

### Statistical analysis

The association between SLE and various cancers was analyzed based on available data, and the pooled SIRs with 95% CIs were utilized to evaluate its efficacy. Heterogeneity was assessed by means of the Chi-square test and *I*^2^ test. If significant heterogeneity (*P* < 0.10 or *I*^2^ > 50%) existed, the random-effects model (the DerSimonian-Laird method) was applied. Otherwise, the fixed-effects model (the Mantel-Haenszel method) was utilized [19]. Moreover, the stability and reliability of the results were determined by sensitivity analysis by deleting one study at a time to reflect the influence of the individual outcomes on the overall outcome. Potential publication bias was accessed by Begg’s funnel plot and Egger’s linear regression test. A *P* value < 0.05 indicated the existence of publication bias [20]. In addition, all the *P* values were adopted by a two-sided test and *P* < 0.05 was considered to be statistically significant. All statistical data were compiled by Stata software (version 12.0; StataCorp LP, College Station, TX, USA) and Microsoft Excel (V.2007; Microsoft Corporation, Redmond, WA, USA).

## Results

### Characteristics of enrolled studies

A total of 2019 relevant articles were identified through a primary literature search using the described search strategy and inclusion/exclusion criteria. After removing duplicates, 1627 records remained. By screening the tittles and abstracts, an additional 639 records were excluded because they were review articles, letters, case-reports, comments, or editorials. Of the remaining 713 full-text articles, 689 were also removed as they were unrelated to SLE or cancers, non-English articles, they had a simple description without comparison, or an absence of key information. Finally, 24 eligible studies were enrolled in this meta-analysis [11, 13–15, 21–40] (Additional file 1: Figure S1).

The detailed characteristics of these 24 eligible studies are summarized in Table 1 and (Additional file 2: Table S1). Specifically, a total of 24 human malignant neoplasms were systematically divided into six systemic groups (lymphatic and hematopoietic cancers, reproductive cancers, urinary cancers, digestive cancers, respiratory cancers, and others). Lymphatic and hematopoietic cancers mainly consisted of non-Hodgkin’s lymphoma, Hodgkin’s lymphoma, multiple myeloma, and leukemia. Reproductive cancers included five cancers (breast cancer, uterus cancer, cervix cancer, ovarian cancer, and vagina/vulva cancer). The urinary cancer group was predominantly made up of renal cancer, prostate cancer, and bladder cancer. Esophagus cancer, gastric cancer, hepatobiliary cancer, pancreatic cancer, and colorectal cancer were involved in the digestive cancers. Lung cancer, oropharynx cancer, and larynx cancer were considered as respiratory cancers. Finally, other cancers were mainly comprised of the following four cancers (cutaneous melanoma, non-melanoma skin cancer, brain cancer, and thyroid cancer).

**Table 1:** Main characteristics of individual studies included in this meta-analysis

First author	Year	Data origin	Calendar period	No.of SLE patients (gender)	SLE diagnosis	Follow-up	NOS scores
Tallbacka [14]	2018	The Helsinki University Central Hospital	1967–1987	205 (182 females and 23 males)	ARA criteria	25.7 years	7
Yun [11]	2017	National Health Insurance System database	2009–2013	17,495 (15,826 females and 1669 males)	NA	NA	8
Azrielant [13]	2017	Clalit Health Services	2013	5018 (all males)	NA	NA	6
Yu [21]	2016	The Taiwan National Health Insurance Research Database (NHIRD)	1997–2010	15,623 (13,693 females and 1930 males)	ACR criteria	124,832.45 person-years	8
Waseem [22]	2015	2006 Medicare claims data	2006	18,423 (all females)	NA	NA	8
Bernatsky [24]	2013	Multi-center	1958–2009	16,409 (90% females)	ACR criteria	7.4 years/121,283 years	8
Dey [23]	2013	The University College London Hospitals Lupus Clinic	1978–2010	595	ACR criteria	14.7 years/8910.51 person-years	6
Hemminki [25]	2012	Swedish Hospital Discharge Register	1964–1986	7624	NA	11.9 years/86,640 person-years	8
Dreyer [26]	2011	Central Population Register	1951–2006	576 (508 females and 68 males)	ACR criteria	13.2 years/7803 years	7
Kang [27]	2010	Kangnam St. Mary’s Hospital	1997–2007	914 (all females)	ACR criteria	5716 person-years	8
Chen [15]	2010	National Health Insurance Research Database	1996–2007	11,763 (10,394 females and 1369 males)	ARA criteria	6.1 years	5
Gadalla [28]	2009	Surveillance, Epidemiology and End Results-Medicare-linked database	1993–2002	340	NA	NA	8
Parikh-Patel [29]	2008	Statewide patient discharge data	1991–2002	30,478 (27,133 females and 3345 males)	NA	5.1 years/157,969 years	6
Tunde [30]	2007	A single center	1970–2004	860 (771 female and 89 male)	ACR criteria	13.4 years	8
Bernatsky [31]	2005	Multi-center	1958–2000	9547 (8607 females and 940 males)	ACR criteria	8.0 years/76,948 person-years	7
Ragnarsson [32]	2003	Icelandic SLE database	1957–2001	238 (213 females and 25 males)	ARA criteria	12.8 years/2774 years	8
Bjornadal [33]	2002	Swedish National Board of Health and Welfare recorded data	1964–1995	5715 (4201 females and 1514 males)	NA	50,246 person-years	8
Cibere [34]	2001	University-based Rheumatic Disease UniT	1975–1994	297 (84% females)	ACR criteria	12 years	7
Sultan [35]	2000	Board of Health and Welfare recorded data	1978–1999	276 (93.5% females)	ARA criteria	4.8 years/1695 years	7
Ramsey-Goldman [36]	1998	NA	NA	616	NA	NA	5
Mellemkjaer [37]	1997	Nationwide Danish Hospital Discharge Register	1977–1989	1585 (1308 females and 277 males)	ACR criteria	6.8 years/10,807 personyears	6
Abu-Shakra [38]	1996	The University of Toronto Lupus Clinic Database	NA	724 (627 females and 97 males)	ACR criteria	7233 person-years	6
Sweeney [39]	1995	NA	NA	219	NA	NA	5
Pettersson [40]	1992	Fourth Department of Medicine, Helsinki University Central Hospital	1967–1987	205 (182 females and 23 males)	ARA criteria	2340 person-years	5

*SLE* systemic lupus erythematosus, *ACR criteria* American College of Rheumatology criteria, *ARA criteria* American Rheumatism Association criteria, *NA* not available.

**Table 2:** Meta-analysis results for included studies of the relationship between SLE and risks of various cancers

Variables	NO.of studies	Effects model	SIR (95%CI)	I-squared (%)	*P* values	Relationship	Publication bias
Overall characteristics							
Overall cancers	10	Random	1.28 (1.16–1.42)	71.9%	<0.001	Increased risks	None
Female	4	Random	1.49 (1.15–1.93)	72.7%	0.012	Increased risks	None
Male	5	Random	1.59 (1.18–2.14)	78.8%	0.001	Increased risks	None
SLE associated with Lymphatic and haematopoietic cancers							
Non-Hodgkin’s lymphoma	11	Random	4.93 (3.81–6.36)	55.2%	0.014	Increased risks	None
Hodgkin’s lymphoma	8	Fixed	2.60 (2.14–3.17)	0.0%	0.660	Increased risks	Existence
Leukemia	10	Fixed	2.01 (1.64–2.47)	24.3%	0.220	Increased risks	None
Multiple myeloma	4	Fixed	1.48 (1.02–2.14)	0.0%	0.744	Increased risks	None
SLE associated with Reproductive cancers							
Breast cancer	19	Random	0.89 (0.77–1.04)	70.1%	< 0.001	No association	None
Uterus cancer	6	Random	0.70 (0.46–1.07)	58.3%	0.035	No association	None
Cervix cancer	11	Fixed	1.56 (1.29–1.88)	4.1%	0.404	Increased risks	None
Ovarian cancer	11	Fixed	0.92 (0.74–1.33)	14.2%	0.309	No association	None
Vagina/vulva cancer	8	Fixed	3.48 (2.69–4.50)	0.0%	0.813	Increased risks	None
SLE associated with Urinary cancers							
Prostate cancer	11	Fixed	0.78 (0.70–0.88)	14.4%	0.307	Decreased risks	None
Renal cancer	6	Random	2.10 (1.11–3.96)	65.2%	0.013	Increased risks	None
Bladder cancer	10	Random	1.86 (1.16–2.99)	75.1%	< 0.001	Increased risks	None
SLE associated with Digestive cancers							
Esophagus cancer	5	Fixed	1.64 (1.43–1.87)	0.0%	0.725	Increased risks	None
Gastric cancer	8	Fixed	1.31 (1.04–1.63)	0.0%	0.789	Increased risks	None
Hepatobiliary cancer	11	Random	2.37 (1.67–3.38)	50.4%	0.028	Increased risks	None
Pancreatic cancer	9	Fixed	1.24 (0.97–1.60)	6.2%	0.384	No association	None
Colorectal cancer	13	Fixed	0.97 (0.85–1.09)	0.0%	0.907	No association	None
SLE associated with Respiratory cancers							
Lung cancer	15	Random	1.62 (1.40–1.87)	46.0%	0.026	Increased risks	None
Oropharynx cancer	5	Fixed	1.52 (1.00–2.30)	0.0%	0.721	Increased risks	None
Larynx cancer	4	Fixed	2.90 (1.82–4.62)	15.3%	0.315	Increased risks	None
SLE associated with Other cancers							
Cutaneous melanoma	6	Fixed	0.72 (0.56–0.93)	0.0%	0.424	Decreased risks	None
Non-melanoma skin cancer	4	Fixed	1.41 (1.07–1.87)	28.7%	0.240	Increased risks	None
Brain cancer	6	Fixed	1.08 (0.64–1.81)	0.0%	0.765	No association	None
Thyroid cancer	7	Fixed	1.80 (1.46–2.23)	0.0%	0.795	Increased risks	None

*SIR* standardized incidence rate, *CI* confidence interval

### Overall characteristics

**Fig. 1:**
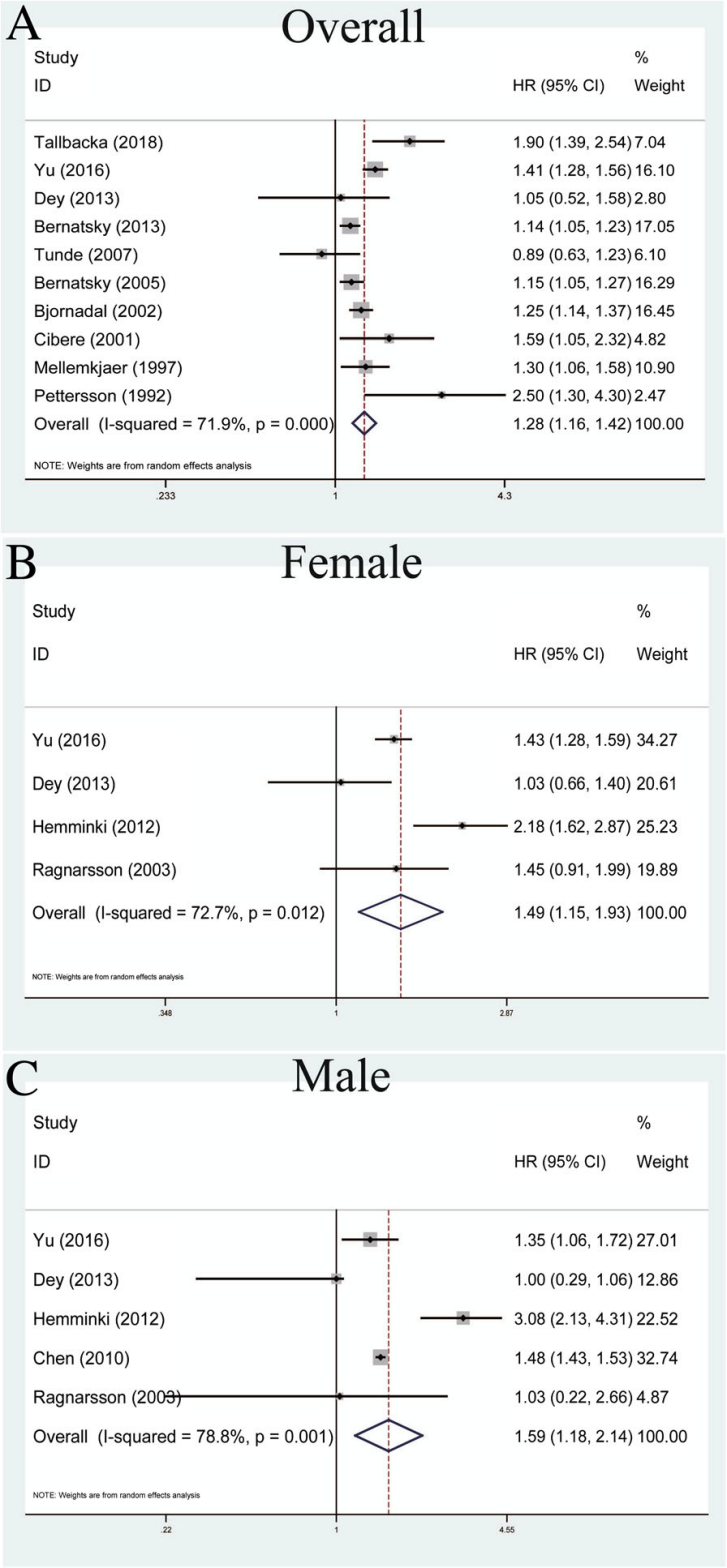
Forest plots of SLE associated with overall characteristics. **a** Overall cancer; **b** the female group; **c** the male group

A total of ten studies contributed to the analysis of SLE and overall cancer incidence within the random-effects model based on the moderate heterogeneity among studies (*P* < 0.001, *I*^*2*^ = 71.9%) (Table 2). Our results indicated that SLE was correlated with increased risk of overall cancers (pooled SIR = 1.28, 95% CI = 1.16–1.42) (Fig. 1a). With regard to the relationship between SLE and gender, the outcomes successfully shed light on SLE being associated with increased risks of both females and males suffering from cancers within the random-effects model (pooled SIR = 1.49, 95% CI = 1.15–1.93, *P* = 0.012, *I*^*2*^ = 72.7% and pooled SIR = 1.59, 95% CI = 1.18–2.14, *P* = 0.001, *I*^*2*^ = 78.8%, respectively) (Fig. 1b, c).

### Association between SLE and lymphatic and hematopoietic cancers

**Fig. 2:**
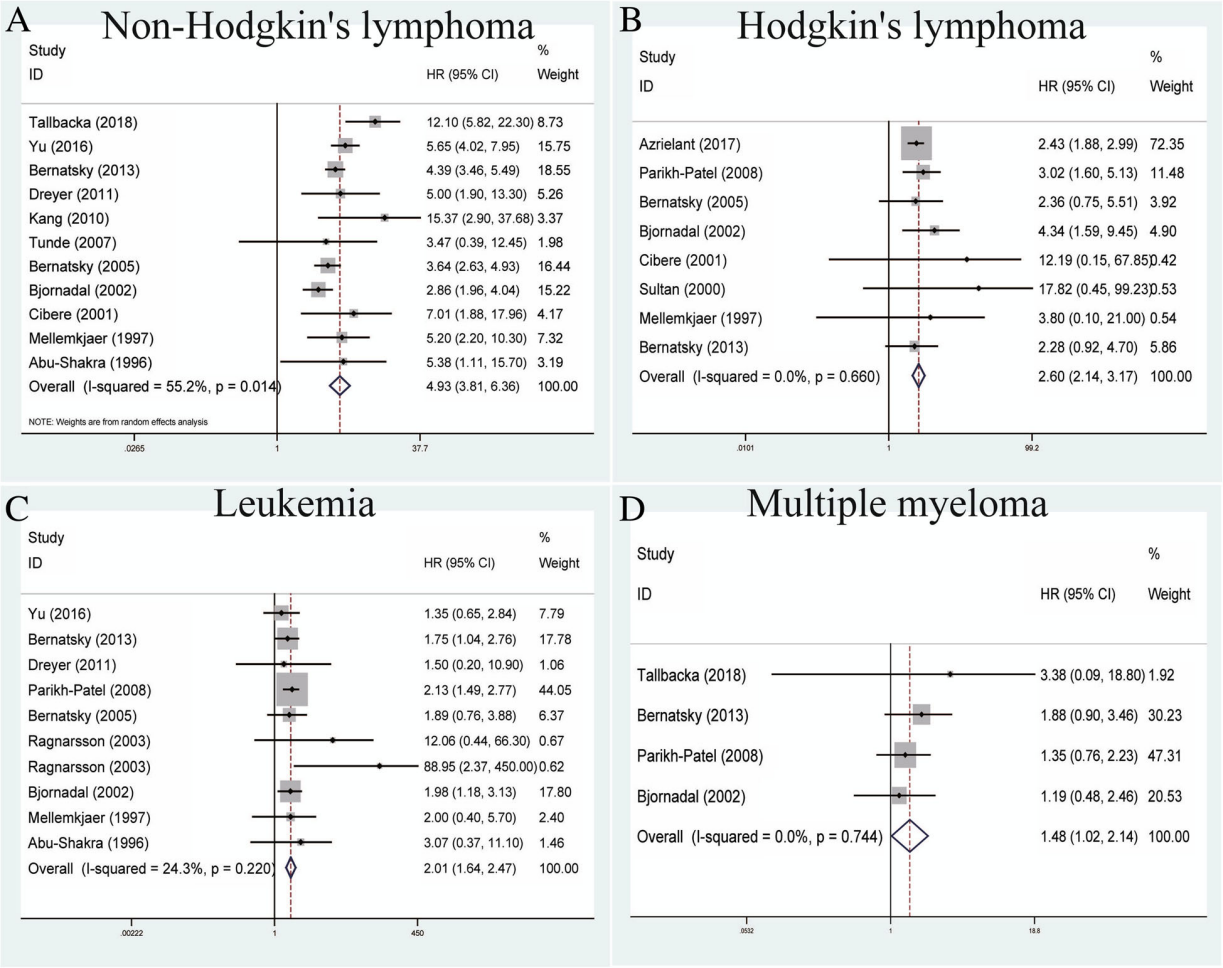
Forest plots of SLE associated with lymphatic and hematopoietic cancers. **a** Non-Hodgkin’s lymphoma; **b** Hodgkin’s lymphoma; **c** leukemia; **d** multiple myeloma

A total of 11 studies contributed to the association between SLE and non-Hodgkin’s lymphoma within the random-effects model based on the moderate heterogeneity among studies (*P* = 0.014, *I*^*2*^ = 55.2%) (Table 2). Our results showed that SLE was correlated with increased risk of non-Hodgkin’s lymphoma (pooled SIR = 4.93, 95% CI = 3.81–6.36) (Fig. 2a). With regard to the relationship between SLE and Hodgkin’s lymphoma, pooled outcomes of eight studies demonstrated that SLE could increase the risk of Hodgkin’s lymphoma within the fixed-effects model (pooled SIR = 2.60, 95% CI = 2.14–3.17, *P* = 0.660, *I*^*2*^ = 0.0%) (Fig. 2b). For leukemia, the analysis of 10 relevant studies showed that SLE was related to an increased risk of leukemia within the fixed-effects model (pooled SIR = 2.01, 95% CI = 1.64–2.47, *P* = 0.220, *I*^*2*^ = 24.3%) (Fig. 2c). Four studies measured the relationship between multiple myeloma and SLE and were analyzed using the fixed-effects model based on no heterogeneity among studies (*P* = 0.744, *I*^*2*^ = 0.0%). Our results also indicated that SLE could increase the risk of multiple myeloma (pooled SIR = 1.48, 95% CI = 1.02–2.14) (Fig. 2d).

### Association between SLE and reproductive cancers

**Fig. 3:**
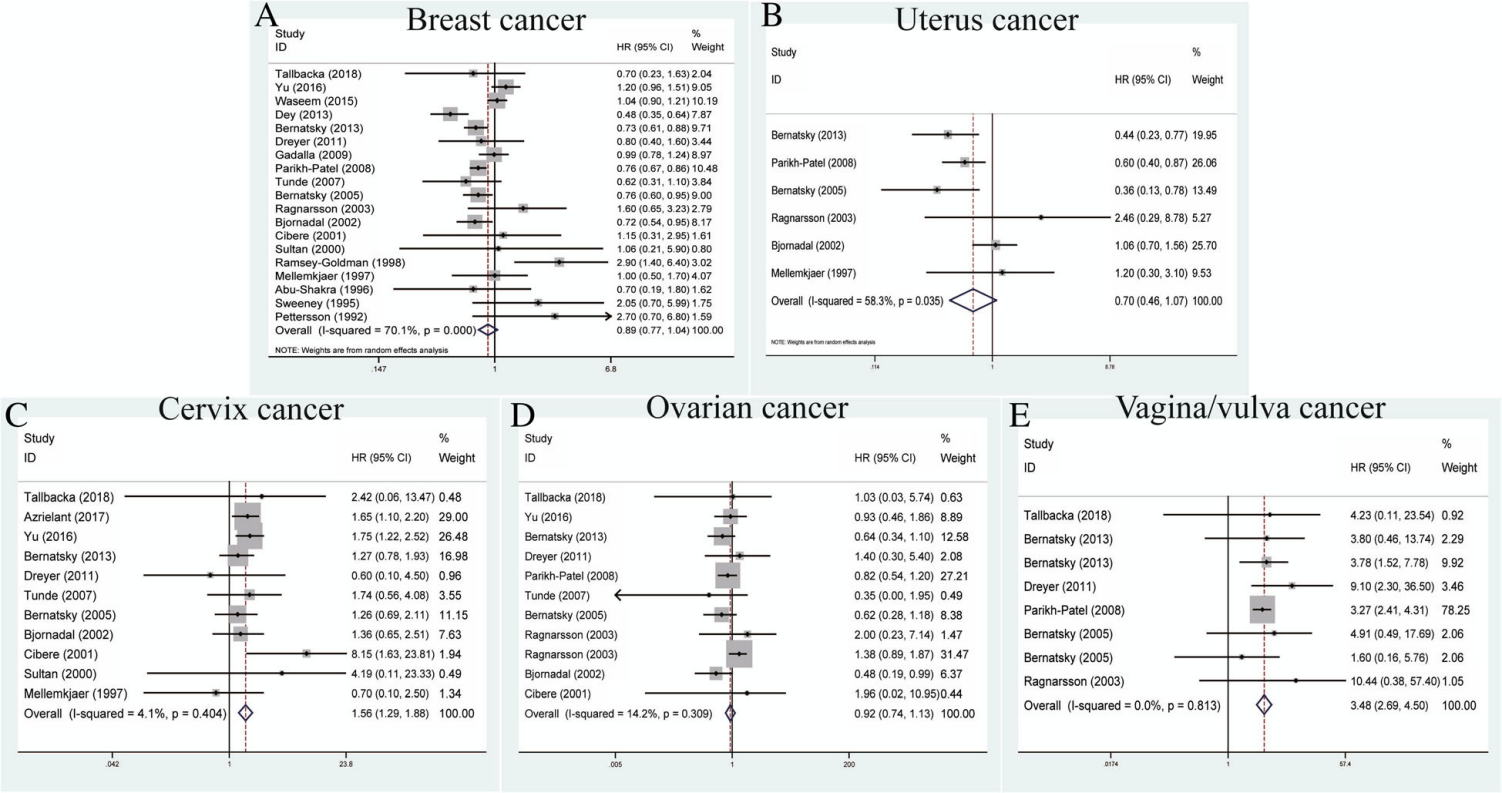
Forest plots of SLE associated with reproductive cancers. **a** Breast cancer; **b** uterus cancer; **c** cervix cancer; **d** ovarian cancer; **e** vagina/vulva cancer

A total of 19 studies contributed to the relationship between SLE and breast cancers within the random-effects model based on the moderate heterogeneity among studies (*P* < 0.001, *I*^*2*^ = 70.1%) (Table 2). Remarkably, our results failed to demonstrate any significant association between them (pooled SIR = 0.89, 95% CI = 0.77–1.04) (Fig. 3a). Similarly, uterus cancers analyzed in six studies showed that SLE was not related to such cancer incidence within the random-effects model (pooled SIR = 0.70, 95% CI = 0.46–1.07, *P* = 0.035, *I*^*2*^ = 58.3%) (Fig. 3b). For cervix cancers, 11 studies showed that SLE was related with increased risk of cervix cancers within the fixed-effects model (pooled SIR = 1.56, 95% CI = 1.29–1.88, *P* = 0.404, *I*^*2*^ = 4.1%) (Fig. 3c). With regard to ovarian cancers, 11 studies failed to display any vital association between them within the fixed-effects model (pooled SIR = 0.92, 95% CI = 0.74–1.33, *P* = 0.309, *I*^*2*^ = 14.2%) (Fig. 3d). Finally, with reference to vagina/vulva cancers, the analysis of eight studies successfully revealed that SLE was correlated with increased risk of vagina/vulva cancers (pooled SIR = 3.48, 95% CI = 2.69–4.50, *P* = 0.813, *I*^*2*^ = 0.0%) (Fig. 3e).

### Association between SLE and urinary cancers

**Fig. 4:**
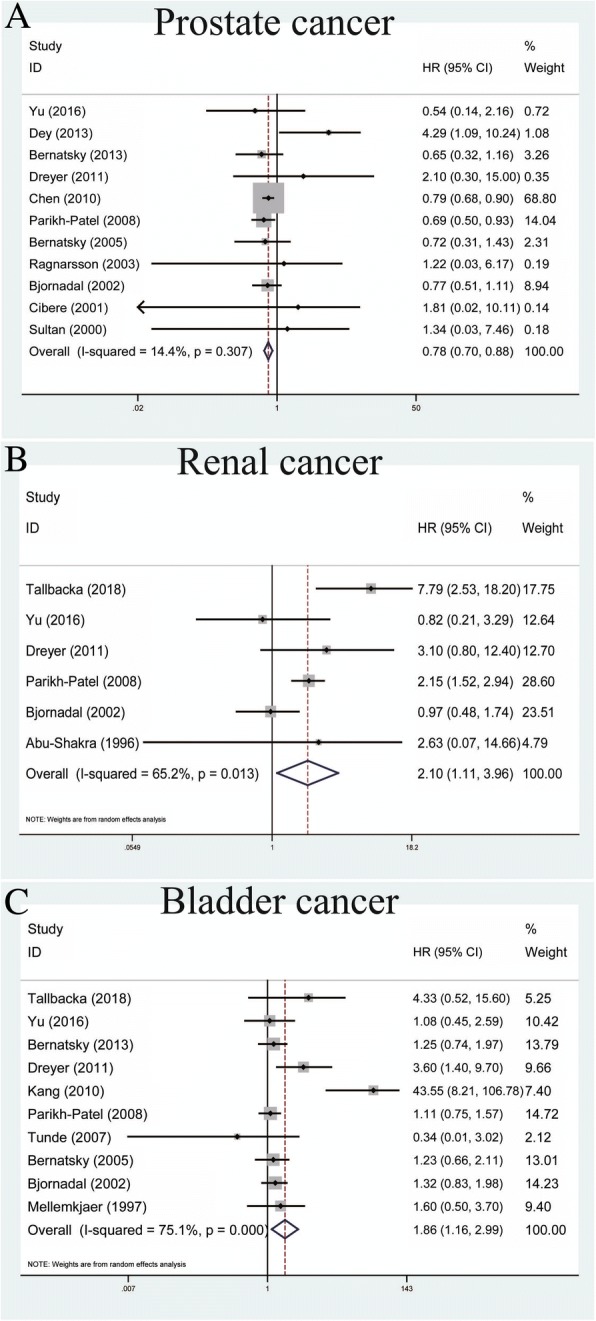
Forest plots of SLE associated with urinary cancers. **a** Prostate cancer; **b** renal cancer; **c** bladder cancer

For urinary cancers, there were 11 studies measuring the association between SLE and prostate cancer within the fixed-effects model based on the low heterogeneity among studies (*P* = 0.307, *I*^*2*^ = 13.4%) (Table 2). Our results revealed that SLE was correlated with decreased risk of prostate cancers (pooled SIR = 0.78, 95% CI = 0.70–0.88) (Fig. 4a). For renal cancer, the analysis of six studies showed that SLE was related to an increased risk of renal cancer within the random-effects model (pooled SIR = 2.10, 95% CI = 1.11–3.96, *P* = 0.013, *I*^*2*^ = 65.2%) (Fig. 4b). With regard to bladder cancer, a total of ten studies showed that SLE was associated with increased risk of bladder cancers within the random-effects model (pooled SIR = 1.86, 95% CI = 1.16–2.99, *P* < 0.001, *I*^*2*^ = 75.1%) (Fig. 4c).

### Association between SLE and digestive cancers

**Fig. 5:**
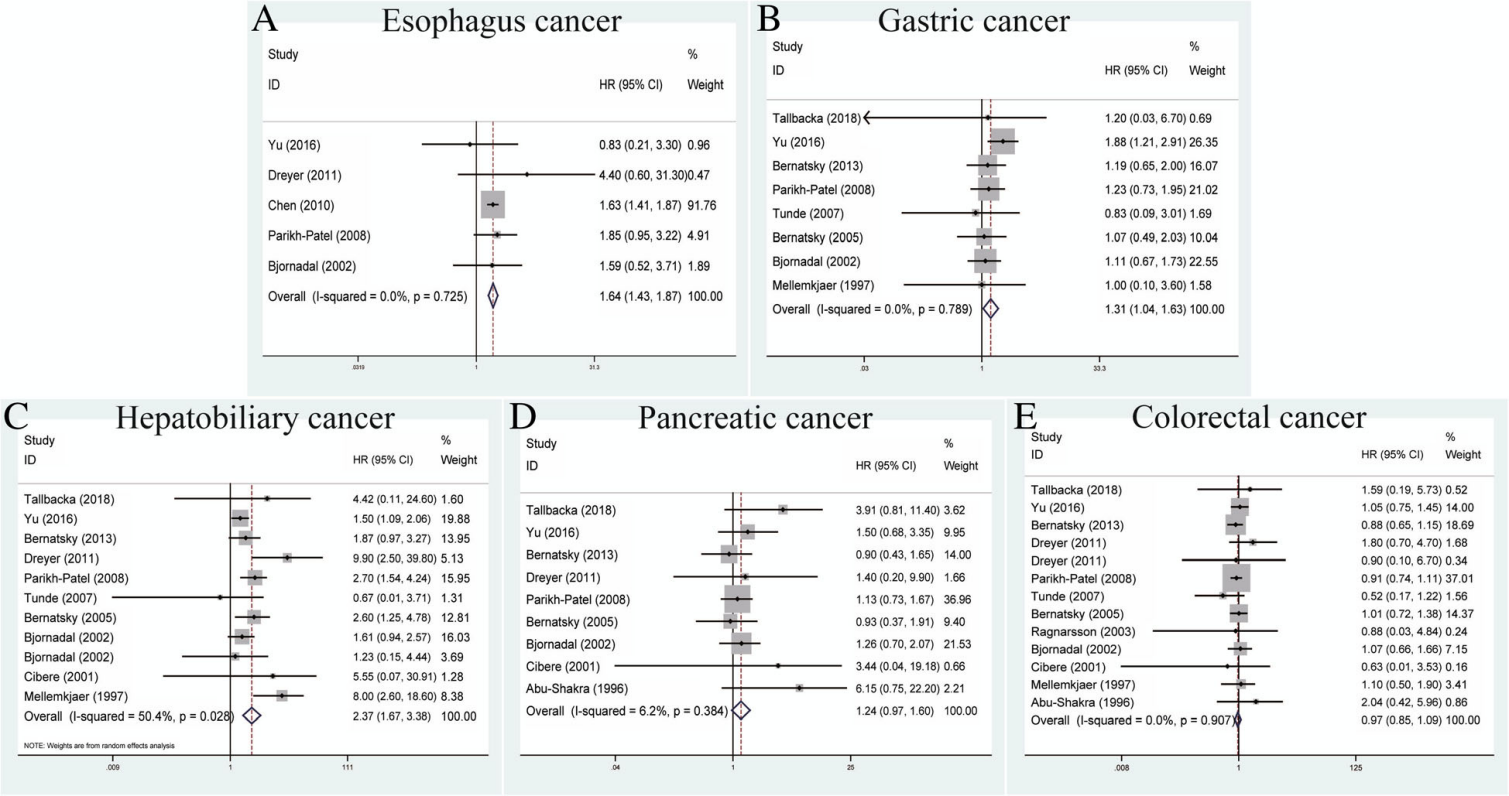
Forest plots of SLE associated with digestive cancers. **a** Esophagus cancer; **b** gastric cancer; **c** hepatobiliary cancer; **d** pancreatic cancer; **e** colorectal cancer

Esophageal cancer was analyzed in a total of five studies to determine its relationship with SLE using the fixed-effects model based on no heterogeneity among studies (*P* = 0.725, *I*^*2*^ = 0.0%) (Table 2). We observed that SLE could increase the risk of esophagus cancer (pooled SIR = 1.64, 95% CI = 1.43–1.87) (Fig. 5a). For gastric cancer, a total of eight studies showed that SLE was related to an increased risk of this cancer within the fixed-effects model (pooled SIR = 1.31, 95% CI = 1.04–1.63, *P* = 0.789, *I*^*2*^ = 0.0%) (Fig. 5b). With regard to hepatobiliary cancers, an analysis of 11 studies showed that SLE was correlated with increased risk within the random-effects model (pooled SIR = 2.37, 95% CI = 1.67–3.38, *P* = 0.028, *I*^*2*^ = 50.4%) (Fig. 5c). Finally, the associations between SLE and pancreatic cancer or colorectal cancer were found to be non-existent using the fixed-effects model (pooled SIR = 1.24, 95% CI = 0.97–1.60, *P* = 0.384, *I*^*2*^ = 6.2%, and pooled SIR = 0.97, 95% CI = 0.85–1.09, *P* = 0.907, *I*^*2*^ = 0.0%, respectively) (Fig. 5d, e).

### Association between SLE and respiratory cancers

**Fig. 6:**
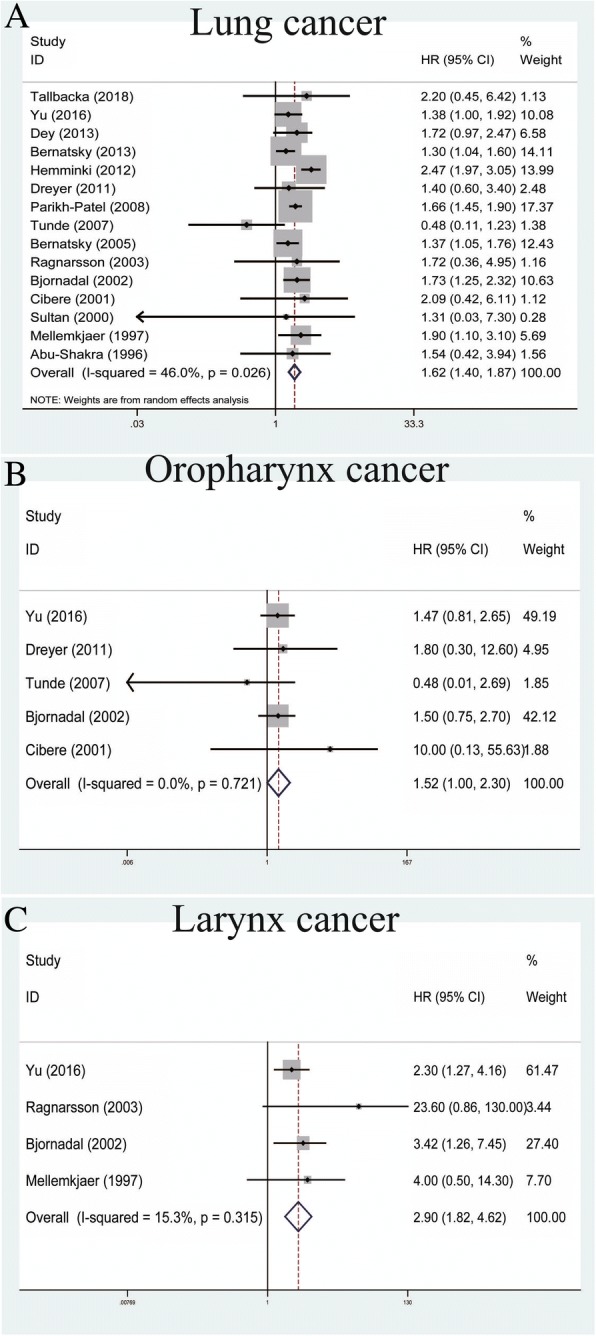
Forest plots of SLE associated with respiratory cancers. **a** Lung cancer; **b** oropharynx cancer; **c** larynx cancer

A total of 15 studies contributed to the analysis of SLE and lung cancer within the random-effects model based on moderate heterogeneity among studies (*P* = 0.026, *I*^*2*^ = 46.0%) (Table 2). The outcomes showed that SLE was correlated with increased risk of lung cancers (pooled SIR = 1.62, 95% CI = 1.40–1.87) (Fig. 6a). For oropharynx cancer, a total of five studies showed that SLE was connected with an increased risk of oropharynx cancer within the fixed-effects model (pooled SIR = 1.52, 95% CI = 1.00–2.30, *P* = 0.721, *I*^*2*^ = 0.0%) (Fig. 6b). Finally, with regard to larynx cancer, an analysis of four studies indicated that SLE was correlated with an increased risk within the fixed-effects model (pooled SIR = 2.90, 95% CI = 1.82–4.62, *P* = 0.315, *I*^*2*^ = 15.3%) (Fig. 6c).

### Association between SLE and other cancers

**Fig. 7:**
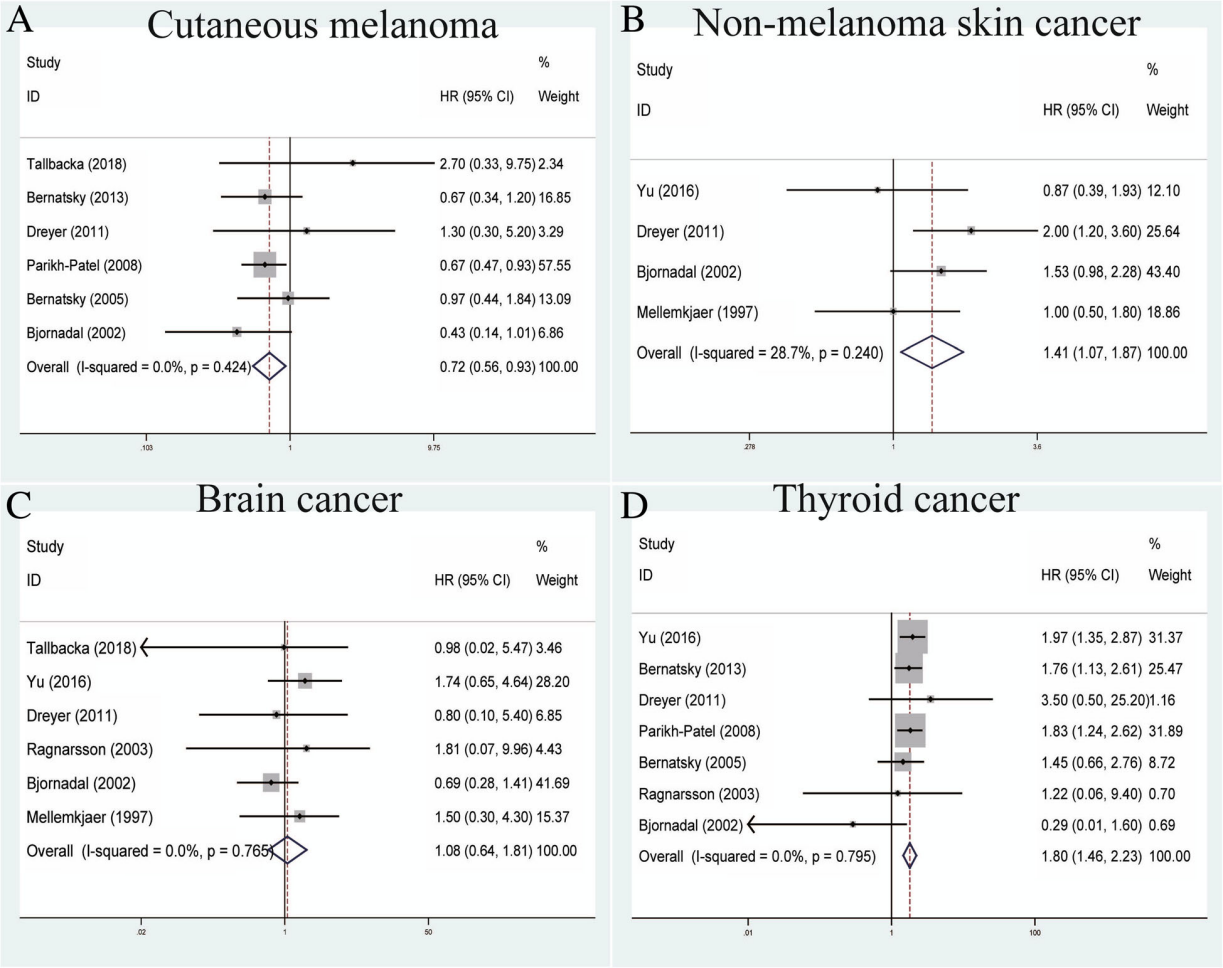
Forest plots of SLE associated with other cancers. **a** Cutaneous melanoma; **b** non-melanoma skin cancer; **c** brain cancer; **d** thyroid cancer

We analyzed six studies measuring the correlation between SLE and cutaneous melanoma within the fixed-effects model based on no heterogeneity among studies (*P* = 0.424, *I*^*2*^ = 0.0%) (Table 2). Our results showed that SLE was correlated with a decreased risk of cutaneous melanoma (pooled SIR = 0.72, 95% CI = 0.56–0.93) (Fig. 7a). For non-melanoma skin cancers, four studies indicated that SLE could increase its risk within the fixed-effects model (pooled SIR = 1.41, 95% CI = 1.07–1.87, *P* = 0.240, *I*^*2*^ = 28.7%) (Fig. 7b). Interestingly, brain cancer failed to demonstrate any significant association with SLE in six studies using the fixed-effects model (pooled SIR = 1.08, 95% CI = 0.64–1.81, *P* = 0.765, *I*^*2*^ = 0.0%) (Fig. 7c). For the association between SLE and thyroid cancer, a total of seven studies indicated that SLE was associated with an increased risk of thyroid cancer (pooled SIR = 1.80, 95% CI = 1.46–2.23, *P* = 0.795, *I*^*2*^ = 0.0%) (Fig. 7d).

### Sensitivity analysis

Sensitivity analysis was conducted by deleting a single study each time to observe the influence of the individual outcome on the overall analysis. As indicated by the results of analysis, most of the pooled SIRs with 95% CIs were not remarkably influenced by any individual study. This demonstrated the stability and reliability of our results (Additional file 3: Figure S2). However, in the analysis of male category, the study by Chen et al. [15] was found to significantly influence the estimated pooled SIR (Additional file 3: Figure S2C).

### Publication bias

Potential publication bias was assessed by Begg’s funnel plot and Egger’s linear regression test. A *P* value of < 0.05 indicated the existence of publication bias (Additional file 4: Figure S3). As indicated by our results, we found that most of the *P* values of Begg’s and Egger’s test were above 0.05, indicating no significant publication bias except for those results outlined in Additional file 4: Figure S3E.

## Discussion

To the best of our knowledge, this is the first and largest systematic evaluation to reveal the relationship between SLE and the development of cancer risk. The outcomes successfully shed light on SLE increasing the risks of overall cancer, cancer risk in both genders, non-Hodgkin’s lymphoma, Hodgkin’s lymphoma, leukemia, multiple myeloma, cervix, vagina/vulva, renal, bladder, esophagus, gastric, hepatobiliary, lung, oropharynx, larynx, non-melanoma skin, and thyroid cancers. Moreover, SLE could decrease the risks of prostate cancer and cutaneous melanoma. In addition, no significant associations were revealed between SLE and breast, uterus, ovarian, pancreatic, colorectal, or brain cancers.

In line with previous research, Ni et al. demonstrated that SLE patients were at increased risk of developing lung or liver cancers and a decreased risk of suffering from prostate cancer [41]. Similarly, Rezaieyazdi et al. suggested there was no direct association between SLE and risk of breast cancer incidence [16]. Inconsistent with our results, Bernatsky et al. supported a decreased risk of breast, ovarian, and endometrial cancers in SLE [42]. Huang et al. also indicated that SLE was not associated with the risk of bladder cancer [43], whereas the outcomes in our meta-analysis showed a positive association between SLE and bladder cancer. The reason for this might be that their study was composed of diminutive sample sizes without sufficient statistical power. Moreover, our results reconfirmed the deterioration of bladder carcinoma in association with SLE treatment observed in several case series [44, 45].

Interestingly, our results indicated that SLE was correlated with an increased risk in overall cancers and, meanwhile, 16 of 24 analyzed cancers were positively associated with SLE; only prostate cancer and cutaneous melanoma showed a negative association with SLE. Mok and Lau suggested that a relatively lower level of testosterone, a critical risk factor for prostate cancer, might account for the decreased risk of prostate cancer in SLE compared with males without SLE [46]. Moreover, several important co-stimulatory molecules had been demonstrated to play crucial roles in both the pathogenesis of SLE and carcinogenesis, such as OX40L and CTLA4 [47, 48]. Hence, we hypothesize that testosterone and several co-stimulatory molecules in these two cancers might reverse the oncogenic role of SLE. More attention should be paid to the underlying potential mechanisms between SLE and cancer risk in further studies.

Several potential mechanisms could account for cancer development in SLE patients. These patients, by virtue of their disease, have basic defects in immune cell function, resulting in immune dysregulation which might prevent aberrant cells from being removed and eventually contributing to increased cancer risk [49]. On the other hand, drugs for immunosuppressive therapy could also potentiate immune dysregulation and lead to further increased risks for developing cancer [50]. Other studies also reported the existence of several important co-stimulatory molecules, including OX40L and CTLA4, which could play crucial roles in both the pathogenesis of SLE and carcinogenesis [47, 48]. Additionally, as a pivotal regulatory element of the immune response magnitude, CTLA4 could be considered as a two-sided knife which predisposes individuals to tumor growth and/or progression under extraordinary expression and accelerates the formation and/or manifestation of inflammatory autoimmune disorders under compromised expression. An association between CTLA4 and SLE not only targets position +49 at the leader peptide but also screens the other single nucleotide polymorphic variants (SNPs) located at the regulatory region and the 3’ untranslated region (UTR). However, this hypothesis requires further investigation of the association between the CTLA4 gene at position +49A/G and SLE because of other relevant studies with inconsistent results.

Several risk factors should also be taken into consideration. Smoking could be regarded as a significant etiologic agent for cancer development in SLE. Compared with those who did not smoke, the lung cancer risk of lupus patients who smoked was found to be increased almost four-fold (adjusted hazard ratio (HR) = 3.6, 95% CI = 1.32–9.83). This underlined once again the universal importance of smoking cessation, particularly in chronic autoimmune disorders such as SLE [51]. Bernatsky et al. put forward the hypotheses that breast cancer risk in SLE might be influenced by autoantibody profiles or drug exposures, such as nonsteroidal anti-inflammatory drugs and antimalarial drugs, although no definite associations were ultimately revealed [52]. As for the increased incidence rate of non-Hodgkin’s lymphoma in patients with SLE, Kang et al. proposed that abnormal B-cell function and the use of immunosuppressive agents might lead to lymphoma by direct mutagenesis or by disturbing immune surveillance [27]; other factors include age, underlying genetic factors, environmental triggers.

Notably, as displayed in Table 1, nine enrolled studies including several of the biggest ones did not report diagnostic criteria for SLE. Among these studies, most of them utilized the research databases such as the Center for Primary Health Care Research, the National Health Insurance Claims Database, and Patient Discharge Dataset, which recorded complete data on all discharges with dates of hospitalization and diagnoses, the International Classification of Diseases codes, and so on. Therefore, these studies relied on the diagnosis having been recorded correctly and were easily associated with the selection bias of patient inclusion. Hence, further confirmation on diagnostic criteria were required to minimize these issues. Furthermore, repeated analysis was conducted to include only those papers in which SLE was diagnosed according to accepted criteria. As detailed in Additional file 5: Table S2, most of our results were consistent, except for renal cancer, oropharynx cancer, cutaneous melanoma, and non-melanoma skin cancer. Our re-analysis indicated that no significant associations were revealed between SLE and these four cancers. More relevant studies with larger sample sizes are required to verify our findings. Results from sensitivity analysis and publication bias should also be discussed. The pooled SIRs with 95% CIs were not significantly influenced by individual studies, suggesting stability of our results (Additional file 3: Figure S2C). For the male category, the study by Chen et al. [15] was found to significantly influence the estimated pooled SIR. Similarly, the *P* values of Begg’s and Egger’s test were all above 0.05, indicating the absence of significant publication bias, except as indicated in Additional file 4: Figure S3E, where a *P* value for Begg’s test was 0.083 and a *P* value for Egger’s test was 0.036, indicating the existence of publication bias. When considering these two aspects, the outcomes should be interpreted with caution.

The strengths of this study were mainly the well-designed methodology of the meta-analysis and the enrollment of all eligible studies, thus providing sufficient statistical power to draw a comprehensive conclusion. Finally, heterogeneity in this study remained low to moderate, even without heterogeneity. Nonetheless, several potential limitations should also be acknowledged. Firstly, the article language was restricted to English, and some relevant articles written in other languages might have been missed. Moreover, although most of our results indicated no significant publication bias, some small negative studies are less likely to be published. Secondly, due to the limited data on this topic, some confounding factors (such as age, sex, and environmental triggers) were not fully clarified, which could result in an inaccurate estimation of their true relationship. Finally, due to insufficient data extracted from primary articles, subgroup analyses were not performed on factors such as ethnicity, alcohol use, and smoking.

## Conclusions

Taken together, our results shed light on SLE being associated with increased risks of overall cancer, females or males suffering from cancers, non-Hodgkin’s lymphoma, Hodgkin’s lymphoma, leukemia, multiple myeloma, cervix, vagina/vulva, renal, bladder, esophagus, gastric, hepatobiliary, lung, oropharynx, larynx, non-melanoma skin, and thyroid cancers, and decreased risks of prostate cancer and cutaneous melanoma. Moreover, no significant associations were revealed between SLE and breast, uterus, ovarian, pancreatic, colorectal, or brain cancers. Despite the aforementioned limitations, these outcomes provide a fairly valid and generalizable description of the occurrence of cancers in SLE. Future high-quality research is required to verify our findings and this should pay more attention to the underlying mechanisms between SLE and cancers risks.

## Additional files

Additional file 1: **Figure S1.** Flow diagram of the literature selection process. (PDF 353 kb)

Additional file 2: **Table S1.** SIRs with 95%CIs and Observed/Expected events of individual studies enrolled in this study. (DOCX 37.2 kb)

Additional file 3: **Figure S2.** Sensitivity analysis of each included study; (A) The overall cancer; (B) The female group; (C) The male group; (D) Non-Hodgkin's lymphoma; (E) Hodgkin's lymphoma; (F) Leukemia; (G) Multiple myeloma; (H) Breast cancer; (I) Uterus cancer; (J) Cervix cancer; (K) Ovarian cancer; (L) Vagina/vulva cancer; (M) Prostate cancer; (N) Renal cancer; (O) Bladder cancer; (P) Esophagus cancer; (Q) Gastric cancer; (R) Hepatobiliary cancer; (S) Pancreatic cancer; (T) Colorectal cancer; (U) Lung cancer; (V) Oropharynx cancer; (W) Larynx cancer; (X) Cutaneous melanoma; (Y) Non-melanoma skin cancer; (Z) Brain cancer; (β) Thyroid cancer. (PDF 3250 kb)

Additional file 4: **Figure S3.** Begg’s funnel plots of the publication bias; (A) The overall cancer; (B) The female group; (C) The male group; (D) Non-Hodgkin's lymphoma; (E) Hodgkin's lymphoma; (F) Leukemia; (G) Multiple myeloma; (H) Breast cancer; (I) Uterus cancer; (J) Cervix cancer; (K) Ovarian cancer; (L) Vagina/vulva cancer; (M) Prostate cancer; (N) Renal cancer; (O) Bladder cancer; (P) Esophagus cancer; (Q) Gastric cancer; (R) Hepatobiliary cancer; (S) Pancreatic cancer; (T) Colorectal cancer; (U) Lung cancer; (V) Oropharynx cancer; (W) Larynx cancer; (X) Cutaneous melanoma; (Y) Non-melanoma skin cancer; (Z) Brain cancer; (β) Thyroid cancer. (PDF 1860 kb)

Additional file 5: **Table S2.** Meta-analysis results for included studies diagnosed according to accepted criteria of SLE. (DOCX 2320 kb)

## Abbreviations

CI: Confidence interval
MeSH: Medical Subject Headings
NOS: Newcastle-Ottawa Scale
PRISMA: Preferred Reporting Items for Systematic Reviews and Meta-Analyses
SIR: Standardized incidence rate
SLE: Systemic lupus erythematosus
