# Supplementary material for: The risks of cancer development in systemic lupus erythematosus (SLE) patients: a systematic review and meta-analysis
Source: Arthritis Res Ther. 2018 Dec 6;20:270. doi: 10.1186/s13075-018-1760-3 (PMC6282326; doi:10.1186/s13075-018-1760-3)
Supplement: Supplementary file 1 — Figure S1. Flow diagram of the literature selection process. (PDF 353 kb) [file 13075_2018_1760_MOESM1_ESM.pdf]

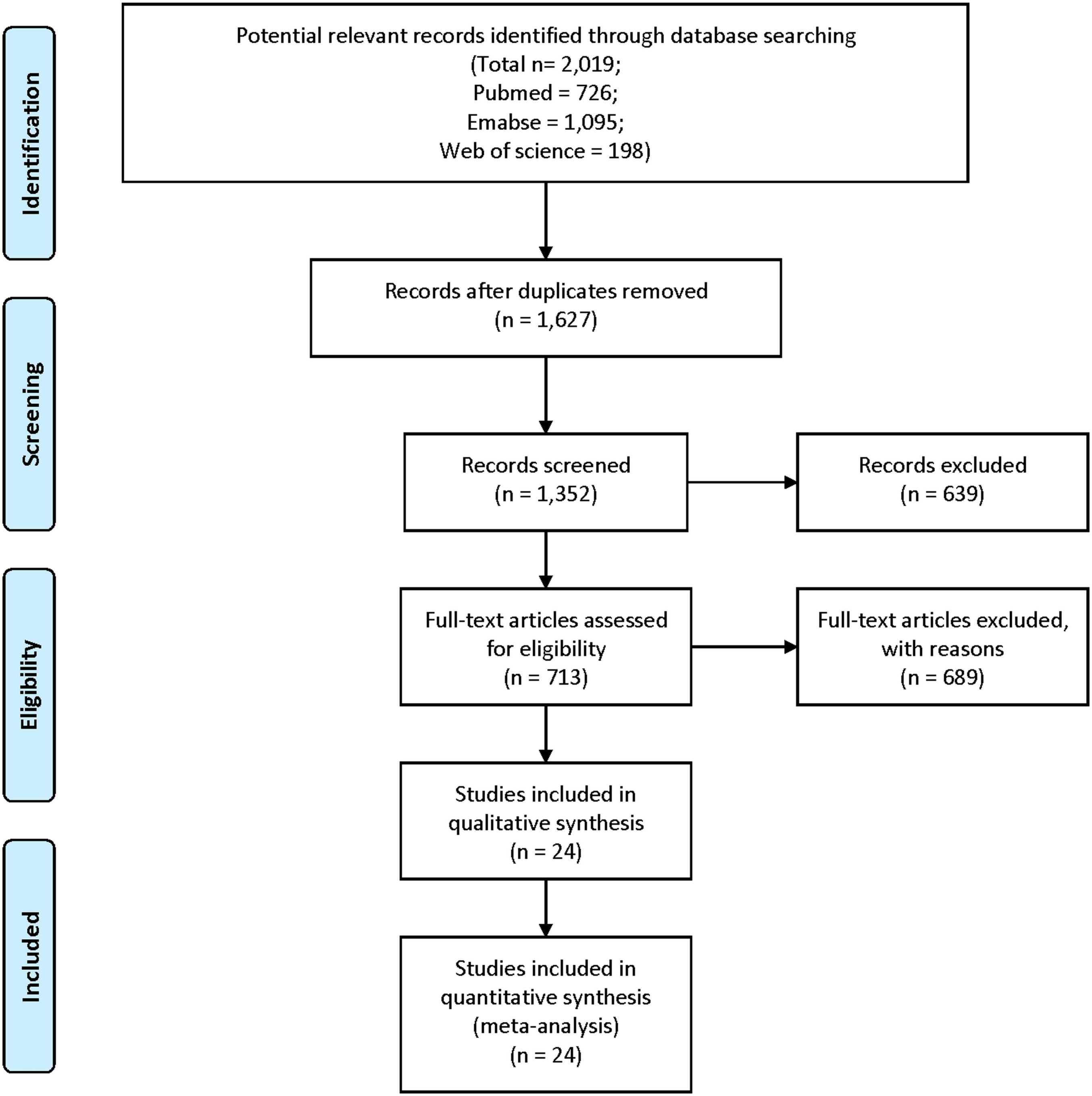
Identification
Screening
Eligibility
Included
Potential relevant records identified through database searching
(Total n= 2,019;
Pubmed = 726;
Emabse = 1,095;
Web of science = 198)
Records after duplicates removed
(n = 1,627)
Records screened
(n = 1,352)
Records excluded
(n = 639)
Full-text articles assessed
for eligibility
(n = 713)
Full-text articles excluded,
with reasons
(n = 689)
Studies included in
qualitative synthesis
(n = 24)
Studies included in
quantitative synthesis
(meta-analysis)
(n = 24)
