# Supplementary material for: The risks of cancer development in systemic lupus erythematosus (SLE) patients: a systematic review and meta-analysis
Source: Arthritis Res Ther. 2018 Dec 6;20:270. doi: 10.1186/s13075-018-1760-3 (PMC6282326; doi:10.1186/s13075-018-1760-3)
Supplement: Supplementary file 2 — Table S1. SIRs with 95%CIs and Observed/Expected events of individual studies enrolled in this study. (DOCX 37.2 kb) [file 13075_2018_1760_MOESM2_ESM.docx]

**Table S1:** SIRs with 95%CIs and Observed/Expected events of individual studies enrolled in this study;

| **Overall cancers** | **First author, year** | Tallbacka,2018 | Yu,2016 | Bernatsky,2013 | Dey,2013 | Chen,2010^★^ | Parikh-Patel,2008^★^ | Tunde,2007 | Bernatsky,2005 | Bjornadal,2002 | Cibere,2001 | Mellemkjaer,1997 |
| --- | --- | --- | --- | --- | --- | --- | --- | --- | --- | --- | --- | --- |
|  | **O/E** | 45/23.68 | 395/299.62 | 644/566.3 | 33/31.5 | 259/147.42 | 1273/1120.8 | 37/41.6 | 431/373.3 | 443/354.0 | 27/16.90 | 102/78.5 |
|  | **SIR(95% CI)** | 1.90(1.39-2.54) | 1.41(1.28-1.56) | 1.14(1.05-1.23) | 1.05(0.52-1.58) | 1.76(1.74-1.79) | 1.14(1.07-1.20) | 0.89(0.626-1.226) | 1.15(1.05-1.27) | 1.25(1.14-1.37) | 1.59(1.05-2.32) | 1.30(1.06-1.58) |
|  | **First author, year** | Pettersson,1992 |  |  |  |  |  |  |  |  |  |  |
|  | **O/E** | 13/5.2 |  |  |  |  |  |  |  |  |  |  |
|  | **SIR(95% CI)** | 2.5(1.3-4.3) |  |  |  |  |  |  |  |  |  |  |
| **Female** | **First author, year** | Yu,2016 | Dey,2013 | Hemminki,2012 | Chen,2010^★^ | Ragnarsson,2003 |  |  |  |  |  |  |
|  | **O/E** | NA | 30/29.0 | 51/23.39 | 221/122.04 | 24/16.59 |  |  |  |  |  |  |
|  | **SIR(95% CI)** | 1.43(1.28-1.59) | 1.03(0.66-1.40) | 2.18(1.62-2.87) | 1.81(1.79-1.83) | 1.45(0.91-1.99) |  |  |  |  |  |  |
| **Male** | **First author, year** | Yu,2016 | Dey,2013 | Hemminki,2012 | Chen,2010 | Ragnarsson,2003 |  |  |  |  |  |  |
|  | **O/E** | NA | 3/3.0 | 34/11.04 | 38/25.67 | 3/2.92 |  |  |  |  |  |  |
|  | **SIR(95% CI)** | 1.35(1.06-1.72) | 1.00(0.29-1.06) | 3.08(2.13-4.31) | 1.48(1.43-1.53) | 1.03(0.22,2.66) |  |  |  |  |  |  |
| **Non-Hodgkin's**  **lymphoma** | **First author, year** | Tallbacka,2018 | Azrielant，2017^★^ | Yu,2016 | Bernatsky,2013 | Dey,2013^★^ | Dreyer,2011 | Kang，2010 | Chen,2010^★^ | Parikh-Patel,2008^★^ | Tunde,2007 | Bernatsky,2005 |
|  | **O/E** | 10/0.82 | NA | NA | 76/17.3 | 1/1.1 | 4/0.8 | 3/0.2 | 24/3.30 | 96/35.1 | 2/0.6 | 42/11.5 |
|  | **SIR(95% CI)** | 12.1(5.82-22.3) | 3.02(2.72-3.33) | 5.65(4.02-7.95) | 4.39(3.46-5.49) | 0.91(0.87-0.95) | 5.0(1.9-13.3) | 15.37(2.90-37.68) | 7.27(6.98-7.57) | 2.74(2.22-3.34) | 3.47(0.387-12.450) | 3.64(2.63-4.93) |
|  | **First author, year** | Bjornadal,2002 | Cibere,2001 | Mellemkjaer,1997 | Abu-Shakra,1996 |  |  |  |  |  |  |  |
|  | **O/E** | 32/11.2 | 4/0.57 | 8/1.5 | 3/0.56 |  |  |  |  |  |  |  |
|  | **SIR(95% CI)** | 2.86(1.96-4.04) | 7.01(1.88-17.96) | 5.2(2.2-10.3) | 5.38(1.11-15.70) |  |  |  |  |  |  |  |
| **Hodgkin's lymphoma** | **First author, year** | Azrielant ，2017 | Bernatsky,2013 | Parikh-Patel,2008 | Bernatsky,2005 | Bjornadal,2002 | Cibere,2001 | Sultan，2000 | Mellemkjaer,1997 |  |  |  |
|  | **O/E** | NA | 7/3.1 | 13/4.3 | 5/2.1 | 6/1.4 | 1/0.08 | 1/0.06 | 1/0.3 |  |  |  |
|  | **SIR(95% CI)** | 2.43(1.88-2.99) | 2.28(0.92-4.70) | 3.02(1.60-5.13) | 2.36(0.75-5.51) | 4.34(1.59-9.45) | 12.19(0.15-67.85) | 17.82(0.45-99.23) | 3.8(0.1-21) |  |  |  |
| **Leukemia** | **First author, year** | Yu,2016 | Bernatsky,2013 | Dreyer,2011 | Chen,2010 | Parikh-Patel,2008 | Bernatsky,2005 | Ragnarsson，2003 | Ragnarsson，2003 | Bjornadal,2002 | Mellemkjaer,1997 | Abu-Shakra,1996 |
|  | **O/E** | NA | 18/10.3 | 1/0.7 | 7/2.65 | 43/20.2 | 7/3.7 | 1/0.08 | 1/0.01 | 18/9.1 | 3/1.5 | 2/0.65 |
|  | **SIR(95% CI)** | 1.35(0.65-2.84) | 1.75(1.04-2.76) | 1.5(0.2-10.9) | 2.64(2.45-2.84) | 2.13(1.49-2.77) | 1.89(0.76-3.88) | 12.06(0.44-66.3) | 88.95(2.37-450) | 1.98(1.18-3.13) | 2.0(0.4-5.7) | 3.07(0.37-11.1) |
| **Multiple myeloma** | **First author, year** | Tallbacka,2018 | Azrielant，2017^★^ | Bernatsky,2013 | Parikh-Patel,2008 | Bjornadal,2002 |  |  |  |  |  |  |
|  | **O/E** | 1/0.30 | NA | 10/5.3 | 15/11.1 | 7/5.9 |  |  |  |  |  |  |
|  | **SIR(95% CI)** | 3.38(0.09-18.8) | 2.57(1.85-3.28) | 1.88(0.90-3.46) | 1.35(0.76-2.23) | 1.19(0.48-2.46) |  |  |  |  |  |  |
| **Breast cancer** | **First author, year** | Tallbacka,2018 | Yu,2016 | Waseem，2015 | Bernatsky,2013 | Dey,2013 | Dreyer,2011 | Chen,2010^★^ | Gadalla，2009 | Parikh-Patel,2008 | Tunde,2007 | Bernatsky,2005 |
|  | **O/E** | 5/7.16 | NA | 416/400 | 114/155.2 | 5/10.5 | 7/9.1 | 45/29.03 | NA | 237/311.9 | 11/17.9 | 73/96.1 |
|  | **SIR(95% CI)** | 0.70(0.23-1.63) | 1.20(0.96-1.51) | 1.04(0.90-1.21) | 0.73(0.61-0.88) | 0.48(0.35-0.64) | 0.8(0.4-1.6) | 1.55(1.51-1.60) | 0.99(0.78-1.24) | 0.76(0.67-0.86) | 0.62(0.307-1.102) | 0.76(0.60-0.95) |
|  | **First author, year** | Ragnarsson，2003 | Bjornadal,2002 | Cibere,2001 | Sultan，2000 | Ramsey-Goldman，1998 | Mellemkjaer,1997 | Abu-Shakra,1996 | Sweeney，1995 | Pettersson，1992 |  |  |
|  | **O/E** | 7/4.38 | 52/72.2 | 4/3.46 | 3/2.83 | 8/2.88 | 14/14.0 | 4/5.71 | NA | 4/1.5 |  |  |
|  | **SIR(95% CI)** | 1.6(0.65-3.23) | 0.72(0.54-0.95) | 1.15(0.31-2.95) | 1.06(0.21-5.9) | 2.9(1.4-6.4) | 1.0(0.5-1.7) | 0.7(0.19-1.8) | 2.05(0.70-5.99) | 2.7(0.7-6.8) |  |  |
| **Uterus cancer** | **First author, year** | Bernatsky,2013 | Chen,2010^★^ | Parikh-Patel,2008 | Bernatsky,2005 | Ragnarsson，2003 | Bjornadal,2002 | Mellemkjaer,1997 |  |  |  |  |
|  | **O/E** | 12/27.2 | 5/3.90 | 29/48.1 | 6/16.9 | 2/0.81 | 26/24.4 | 4/3.3 |  |  |  |  |
|  | **SIR(95% CI)** | 0.44(0.23-0.77 | 1.28(1.17-1.40) | 0.60(0.40-0.87) | 0.36(0.13-0.78) | 2.46(0.29-8.78) | 1.06(0.70-1.56) | 1.2(0.3-3.1) |  |  |  |  |
| **Cervix cancer** | **First author, year** | Tallbacka,2018 | Azrielant，2017 | Yu,2016 | Bernatsky,2013 | Dey,2013^★^ | Dreyer,2011 | Chen,2010^★^ | Parikh-Patel,2008^★^ | Tunde,2007 | Bernatsky,2005 | Bjornadal,2002 |
|  | **O/E** | 1/0.41 | NA | NA | 21/16.6 | 2/0.5 | 1/1.6 | 22/15.83 | 38/69.7 | 5/2.9 | 14/11.1 | 10/7.3 |
|  | **SIR(95% CI)** | 2.42(0.06-13.47) | 1.65(1.10-2.20) | 1.75(1.22-2.52) | 1.27(0.78-1.93) | 4.00(3.50-4.50） | 0.6(0.1-4.5) | 1.39(1.33-1.45) | 0.55(0.39-0.75) | 1.74(0.563-4.080) | 1.26(0.69-2.11) | 1.36(0.65-2.51) |
|  | **First author, year** | Cibere,2001 | Sultan，2000 | Mellemkjaer,1997 |  |  |  |  |  |  |  |  |
|  | **O/E** | 3/0.36 | 1/0.24 | 2/2.8 |  |  |  |  |  |  |  |  |
|  | **SIR(95% CI)** | 8.15(1.63-23.81) | 4.19(0.11-23.33) | 0.7(0.1-2.5) |  |  |  |  |  |  |  |  |
| **Ovarian cancer** | **First author, year** | Tallbacka,2018 | Yu,2016 | Bernatsky,2013 | Dreyer,2011 | Chen,2010^★^ | Parikh-Patel,2008 | Tunde,2007 | Bernatsky,2005 | Ragnarsson，2003 | Ragnarsson，2003 | Bjornadal,2002 |
|  | **O/E** | 1/0.97 | NA | 13/20.2 | 2/1.5 | 3/4.18 | 27/32.8 | 1/2.9 | 9/14.5 | 2/1.00 | 27/19.52 | 7/14.6 |
|  | **SIR(95% CI)** | 1.03(0.03-5.74) | 0.93(0.46-1.86) | 0.64(0.34-1.10) | 1.4(0.3-5.4) | 0.72(0.64-0.80) | 0.82(0.54-1.20) | 0.35(0.005-1.945) | 0.62(0.28-1.18) | 2.00(0.23-7.14) | 1.38(0.89-1.87) | 0.48(0.19-0.99) |
|  | **First author, year** | Cibere,2001 |  |  |  |  |  |  |  |  |  |  |
|  | **O/E** | 1/0.50 |  |  |  |  |  |  |  |  |  |  |
|  | **SIR(95% CI)** | 1.96(0.02-10.95) |  |  |  |  |  |  |  |  |  |  |
| **Vagina/vulva cancer** | **First author, year** | Tallbacka,2018 | Bernatsky,2013 | Bernatsky,2013 | Dreyer,2011 | Chen,2010^★^ | Parikh-Patel,2008 | Bernatsky,2005 | Bernatsky,2005 | Ragnarsson,2003 |  |  |
|  | **O/E** | 1/0.24 | 2/0.5 | 7/1.9 | 2/0.2 | 3/0.63 | 49/15.0 | 2/0.4 | 2/1.3 | 1/0.10 |  |  |
|  | **SIR(95% CI)** | 4.23(0.11-23.54) | 3.80(0.46-13.74) | 3.78(1.52-7.78) | 9.1(2.3-36.5) | 4.76(4.24-5.33) | 3.27(2.41-4.31) | 4.91(0.49-17.69) | 1.60(0.16-5.76) | 10.44(0.38-57.4) |  |  |
| **Prostate cancer** | **First author, year** | Yu,2016 | Bernatsky,2013 | Dey,2013 | Dreyer,2011 | Chen,2010 | Parikh-Patel,2008 | Bernatsky,2005 | Ragnarsson,2003 | Bjornadal,2002 | Cibere,2001 | Sultan，2000 |
|  | **O/E** | NA | 11/16.9 | 3/0.7 | 1/0.5 | 2/2.25 | 43/62.2 | 8/11.1 | 1/0.82 | 28/36.5 | 1/0.55 | 1/0.75 |
|  | **SIR(95% CI)** | 0.54(0.14-2.16) | 0.65(0.32-1.16) | 4.29(1.09-10.24) | 2.1(0.3-15.0) | 0.79(0.68-0.90) | 0.69(0.50-0.93) | 0.72(0.31-1.43) | 1.22(0.03-6.17) | 0.77(0.51-1.11) | 1.81(0.02-10.11) | 1.34(0.03-7.46) |
| **Renal cancer** | **First author, year** | Tallbacka,2018 | Yu,2016 | Dreyer,2011 | Chen,2010^★^ | Parikh-Patel,2008 | Bjornadal,2002 | Abu-Shakra,1996 |  |  |  |  |
|  | **O/E** | 5/0.64 | NA | 2/0.6 | 9/2.25 | 38/17.7 | 11/11.3 | 1/0.38 |  |  |  |  |
|  | **SIR(95% CI)** | 7.79(2.53-18.2) | 0.82(0.21-3.29) | 3.1(0.8-12.4) | 3.99(3.74-4.27) | 2.15(1.52-2.94) | 0.97(0.48-1.74) | 2.63(0.07-14.66) |  |  |  |  |
| **Bladder cancer** | **First author, year** | Tallbacka,2018 | Yu,2016 | Bernatsky,2013 | Dreyer,2011 | Kang，2010 | Chen,2010^★^ | Parikh-Patel,2008 | Tunde，2007 | Bernatsky,2005 | Bjornadal,2002 | Mellemkjaer,1997 |
|  | **O/E** | 2/0.46 | NA | 18/14.4 | 4/1.1 | 3/0.07 | 2/3.05 | 31/28.0 | 1/1.8 | 13/10.5 | 23/17.5 | 5/3.1 |
|  | **SIR(95% CI)** | 4.33(0.52-15.6) | 1.08(0.45-2.59) | 1.25(0.74-1.97) | 3.6(1.4-9.7) | 43.55(8.21-106.78) | 0.66(0.57-0.75) | 1.11(0.75-1.57) | 0.34(0.007-3.024) | 1.23(0.66-2.11) | 1.32(0.83-1.98) | 1.6(0.5-3.7) |
| **Esophagus cancer** | **First author, year** | Yu,2016 | Dreyer,2011 | Chen,2010 | Parikh-Patel,2008 | Bjornadal,2002 |  |  |  |  |  |  |
|  | **O/E** | NA | 1/0.2 | 2/1.23 | 12/6.5 | 5/3.1 |  |  |  |  |  |  |
|  | **SIR(95% CI)** | 0.83(0.21-3.30) | 4.4(0.6-31.3) | 1.63(1.41-1.87) | 1.85(0.95-3.22) | 1.59(0.52-3.71) |  |  |  |  |  |  |
| **Gastric cancer** | **First author, year** | Tallbacka,2018 | Yu,2016 | Bernatsky,2013 | Chen,2010^★^ | Parikh-Patel,2008 | Tunde,2007 | Bernatsky,2005 | Bjornadal,2002 | Mellemkjaer,1997 |  |  |
|  | **O/E** | 1/0.83 | NA | 14/11.8 | 14/6.74 | 18/14.6 | 2/2.4 | 9/8.4 | 19/17.2 | 2/2.0 |  |  |
|  | **SIR(95% CI)** | 1.20(0.03-6.70) | 1.88(1.21-2.91) | 1.19(0.65-2.00) | 2.08(1.97-2.19) | 1.23(0.73-1.95) | 0.83(0.094-3.009) | 1.07(0.49-2.03) | 1.11(0.67-1.73) | 1.0(0.1-3.6) |  |  |
| **Hepatobiliary cancer** | **First author, year** | Tallbacka,2018 | Yu,2016 | Bernatsky,2013 | Dreyer,2011 | Chen,2010^★^ | Parikh-Patel,2008 | Tunde,2007 | Bernatsky,2005 | Bjornadal,2002 | Bjornadal,2002 | Cibere,2001 |
|  | **O/E** | 1/0.23 | NA | 12/6.4 | 2/0.2 | 28/15.32 | 17/6.4 | 1/1.5 | 10/3.8 | 17/10.6 | 2/1.6 | 1/0.18 |
|  | **SIR(95% CI)** | 4.42(0.11-24.6) | 1.50(1.09-2.06) | 1.87(0.97-3.27) | 9.9(2.5-39.8) | 1.83(1.76-1.90) | 2.70(1.54-4.24) | 0.67(0.009-3.709) | 2.60(1.25-4.78) | 1.61(0.94-2.57) | 1.23(0.15-4.44 | 5.55(0.07-30.91) |
|  | **First author, year** | Mellemkjaer,1997 |  |  |  |  |  |  |  |  |  |  |
|  | **O/E** | 5/0.6 |  |  |  |  |  |  |  |  |  |  |
|  | **SIR(95% CI)** | 8.0(2.6-18.6) |  |  |  |  |  |  |  |  |  |  |
| **Pancreatic cancer** | **First author, year** | Tallbacka,2018 | Yu,2016 | Bernatsky,2013 | Dey,2013^★^ | Dreyer,2011 | Chen,2010^★^ | Parikh-Patel,2008 | Bernatsky,2005 | Bjornadal,2002 | Cibere,2001 | Abu-Shakra,1996 |
|  | **O/E** | 3/0.77 | NA | 10/11.2 | 1/0.7 | 1/0.7 | 4/2.00 | 25/22.1 | 7/7.6 | 15/11.9 | 1/0.30 | 2/0.33 |
|  | **SIR(95% CI)** | 3.91(0.81-11.4) | 1.50(0.68-3.35) | 0.90(0.43-1.65) | 1.43(1.32-1.54) | 1.4(0.2-9.9) | 2.00(1.81-2.21) | 1.13(0.73-1.67) | 0.93(0.37-1.91) | 1.26(0.70-2.07 | 3.44(0.04-19.18) | 6.15(0.75-22.20) |
| **Colorectal cancer** | **First author, year** | Tallbacka,2018 | Yu,2016 | Bernatsky,2013 | Dreyer,2011 | Dreyer,2011 | Chen,2010^★^ | Parikh-Patel,2008 | Tunde,2007 | Bernatsky,2005 | Ragnarsson,2003 | Bjornadal,2002 |
|  | **O/E** | 2/1.26 | NA | 51/58.2 | 4/2.3 | 1/1.1 | 14/17.10 | 99/108.2 | 5/9.6 | 40/39.5 | 1/1.14 | 20/18.6 |
|  | **SIR(95% CI)** | 1.59(0.19-5.73) | 1.05(0.75-1.45) | 0.88(0.65-1.15) | 1.8(0.7-4.7) | 0.9(0.1-6.7) | 0.82(0.78-0.86) | 0.91(0.74-1.11) | 0.52(0.169-1.221) | 1.01(0.72-1.38) | 0.88(0.03-4.84) | 1.07(0.66-1.66) |
|  | **First author, year** | Cibere,2001 | Mellemkjaer,1997 | Abu-Shakra,1996 |  |  |  |  |  |  |  |  |
|  | **O/E** | 1/1.57 | 10/9.5 | 3/1.47 |  |  |  |  |  |  |  |  |
|  | **SIR(95% CI)** | 0.63(0.008-3.53 | 1.1(0.5-1.9) | 2.04(0.42-5.96) |  |  |  |  |  |  |  |  |
| **Lung cancer** | **First author, year** | Tallbacka,2018 | Yu,2016 | Bernatsky,2013 | Dey,2013 | Hemminki,2012 | Dreyer,2011 | Chen,2010^★^ | Parikh-Patel,2008 | Tunde,2007 | Bernatsky,2005 | Ragnarsson,2003 |
|  | **O/E** | 3/1.36 | NA | 85/65.5 | 5/2.9 | 85/34.41 | 5/3.5 | 16/13.03 | 218/131.2 | 4/8.3 | 62/45.3 | 3/1.74 |
|  | **SIR(95% CI)** | 2.20(0.45-6.42) | 1.38(1.00-1.92) | 1.30(1.04-1.60) | 1.72(0.97-2.47) | 2.47(1.97-3.05) | 1.4(0.6-3.4) | 1.23(1.17-1.29) | 1.66(1.45-1.90) | 0.48(0.113-1.234) | 1.37(1.05-1.76) | 1.72(0.36-4.95) |
|  | **First author, year** | Bjornadal,2002 | Cibere,2001 | Sultan，2000 | Mellemkjaer,1997 | Abu-Shakra,1996 |  |  |  |  |  |  |
|  | **O/E** | 44/25.5 | 3/1.43 | 1/0.76 | 15/8.0 | 4/2.60 |  |  |  |  |  |  |
|  | **SIR(95% CI)** | 1.73(1.25-2.32) | 2.09(0.42-6.11) | 1.31(0.03-7.30) | 1.9(1.1-3.1) | 1.54(0.42-3.94) |  |  |  |  |  |  |
| **Oropharynx cancer** | **First author, year** | Yu,2016 | Dreyer,2011 | Tunde,2007 | Bjornadal,2002 | Cibere,2001 |  |  |  |  |  |  |
|  | **O/E** | NA | 1/0.6 | 1/2.1 | 11/7.3 | 1/0.10 |  |  |  |  |  |  |
|  | **SIR(95% CI)** | 1.47(0.81-2.65) | 1.8(0.3-12.6) | 0.48(0.006-2.688) | 1.50(0.75-2.70) | 10.00(0.13-55.63) |  |  |  |  |  |  |
| **Larynx cancer** | **First author, year** | Yu,2016 | Ragnarsson,2003 | Bjornadal,2002 | Mellemkjaer,1997 |  |  |  |  |  |  |  |
|  | **O/E** | NA | 1/0.04 | 6/1.8 | 2/0.5 |  |  |  |  |  |  |  |
|  | **SIR(95% CI)** | 2.30(1.27-4.16) | 23.60(0.86-130) | 3.42(1.26-7.45) | 4.0(0.5-14.3) |  |  |  |  |  |  |  |
| **Melanoma of skin** | **First author, year** | Tallbacka,2018 | Bernatsky,2013 | Dreyer,2011 | Parikh-Patel,2008 | Bernatsky,2005 | Bjornadal,2002 |  |  |  |  |  |
|  | **O/E** | 2/0.74 | 11/16.3 | 2/1.6 | 37/54.9 | 9/9.3 | 5/11.6 |  |  |  |  |  |
|  | **SIR(95% CI)** | 2.70(0.33-9.75) | 0.67(0.34-1.20) | 1.3(0.3-5.2) | 0.67(0.47-0.93) | 0.97(0.44-1.84) | 0.43(0.14-1.01) |  |  |  |  |  |
| **Non-melanoma**  **skin cancer** | **First author, year** | Tallbacka,2018^★^ | Yu,2016 | Dreyer,2011 | Bjornadal,2002 | Mellemkjaer,1997 |  |  |  |  |  |  |
|  | **O/E** | 10/0.82 | NA | 12/5.9 | 24/15.7 | 10/10.1 |  |  |  |  |  |  |
|  | **SIR(95% CI)** | 12.1(5.82-22.3) | 0.87(0.39-1.93) | 2.0(1.2-3.6) | 1.53(0.98-2.28) | 1.0(0.5-1.8) |  |  |  |  |  |  |
| **Brain cancer** | **First author, year** | Tallbacka,2018 | Yu,2016 | Dreyer,2011 | Chen,2010^★^ | Ragnarsson,2003 | Bjornadal,2002 | Mellemkjaer,1997 |  |  |  |  |
|  | **O/E** | 1/1.02 | NA | 1/1.3 | 5/1.52 | 1/0.55 | 7/10.2 | 3/2.0 |  |  |  |  |
|  | **SIR(95% CI)** | 0.98(0.02-5.47) | 1.74(0.65-4.64) | 0.8(0.1-5.4) | 3.30(3.00-3.59) | 1.81(0.07-9.96) | 0.69(0.28-1.41) | 1.5(0.3-4.3) |  |  |  |  |
| **Thyroid cancer** | **First author, year** | Yun,2017^★^ | Yu,2016 | Bernatsky,2013 | Dreyer,2011 | Chen,2010^★^ | Parikh-Patel,2008 | Bernatsky,2005 | Ragnarsson,2003 | Bjornadal,2002 |  |  |
|  | **O/E** | NA | NA | 24/13.7 | 1/0.3 | 14/6.26 | 30/16.4 | 9/6.2 | 1/0.58 | 1/3.5 |  |  |
|  | **SIR(95% CI)** | 1.40(1.22-1.60) | 1.97(1.35-2.87) | 1.76(1.13-2.61) | 3.5(0.5-25.2) | 2.24(2.12-2.36) | 1.83(1.24-2.62) | 1.45(0.66-2.76) | 1.22(0.06-9.40) | 0.29(0.01-1.60) |  |  |

O/E: Observed/Expected; SIR: standardized incidence rate; CI: confidence interval;

^★^Articles were ultimately deleted, based on the results of sensitivity analysis.
