# Supplementary figures and images for: The risks of cancer development in systemic lupus erythematosus (SLE) patients: a systematic review and meta-analysis
Source: Arthritis Res Ther. 2018 Dec 6;20:270. doi: 10.1186/s13075-018-1760-3 (PMC6282326; doi:10.1186/s13075-018-1760-3)

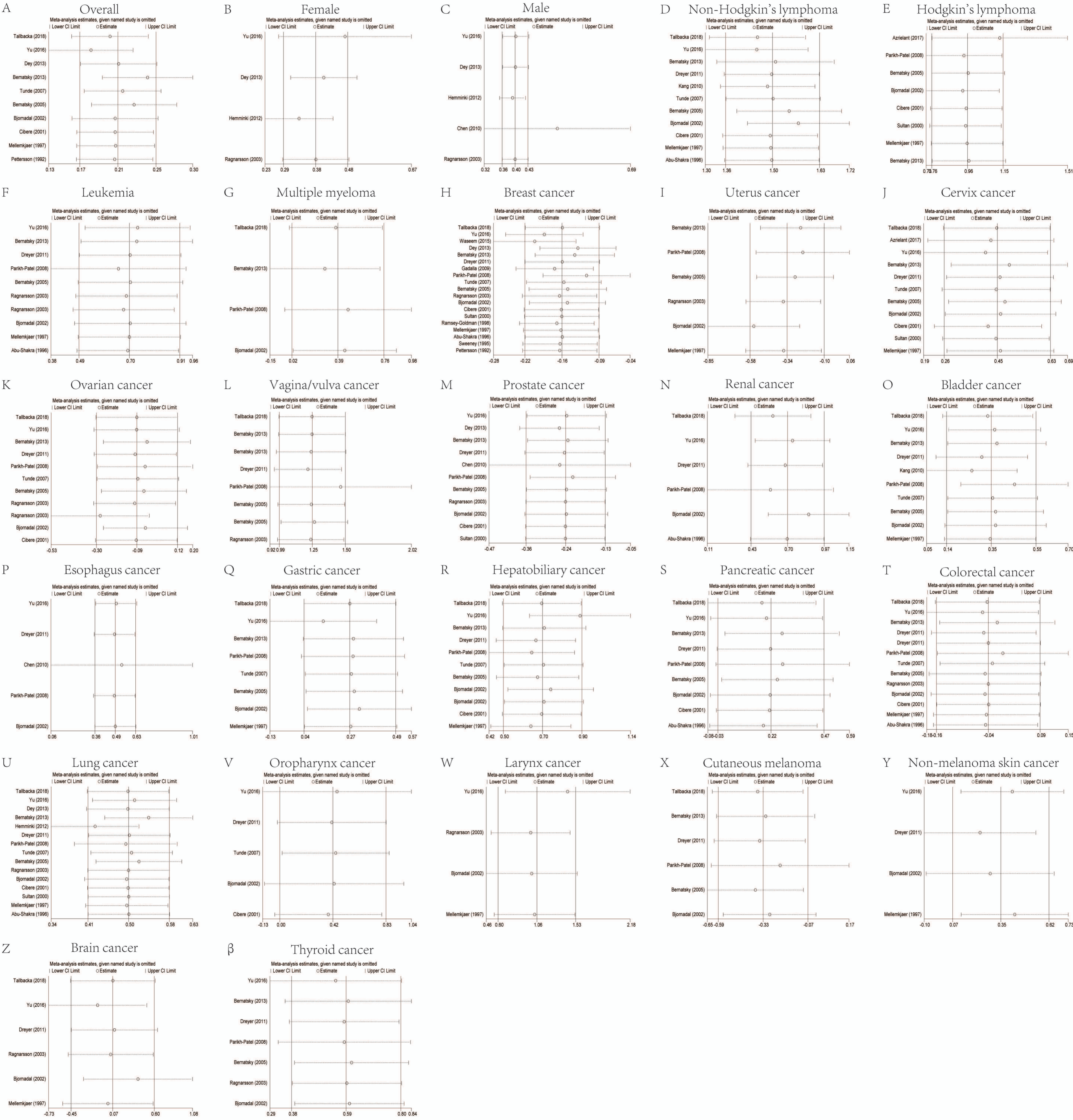

Supplement: Supplementary file 3 — Figure S2. Sensitivity analysis of each included study; (A) The overall cancer; (B) The female group; (C) The male group; (D) Non-Hodgkin's lymphoma; (E) Hodgkin's lymphoma; (F) Leukemia; (G) Multiple myeloma; (H) Breast cancer; (I) Uterus cancer; (J) Cervix cancer; (K) Ovarian cancer; (L) Vagina/vulva cancer; (M) Prostate cancer; (N) Renal cancer; (O) Bladder cancer; (P) Esophagus cancer; (Q) Gastric cancer; (R) Hepatobiliary cancer; (S) Pancreatic cancer; (T) Colorectal cancer; (U) Lung cancer; (V) Oropharynx cancer; (W) Larynx cancer; (X) Cutaneous melanoma; (Y) Non-melanoma skin cancer; (Z) Brain cancer; (β) Thyroid cancer. (PDF 3250 kb) [file 13075_2018_1760_MOESM3_ESM.pdf]

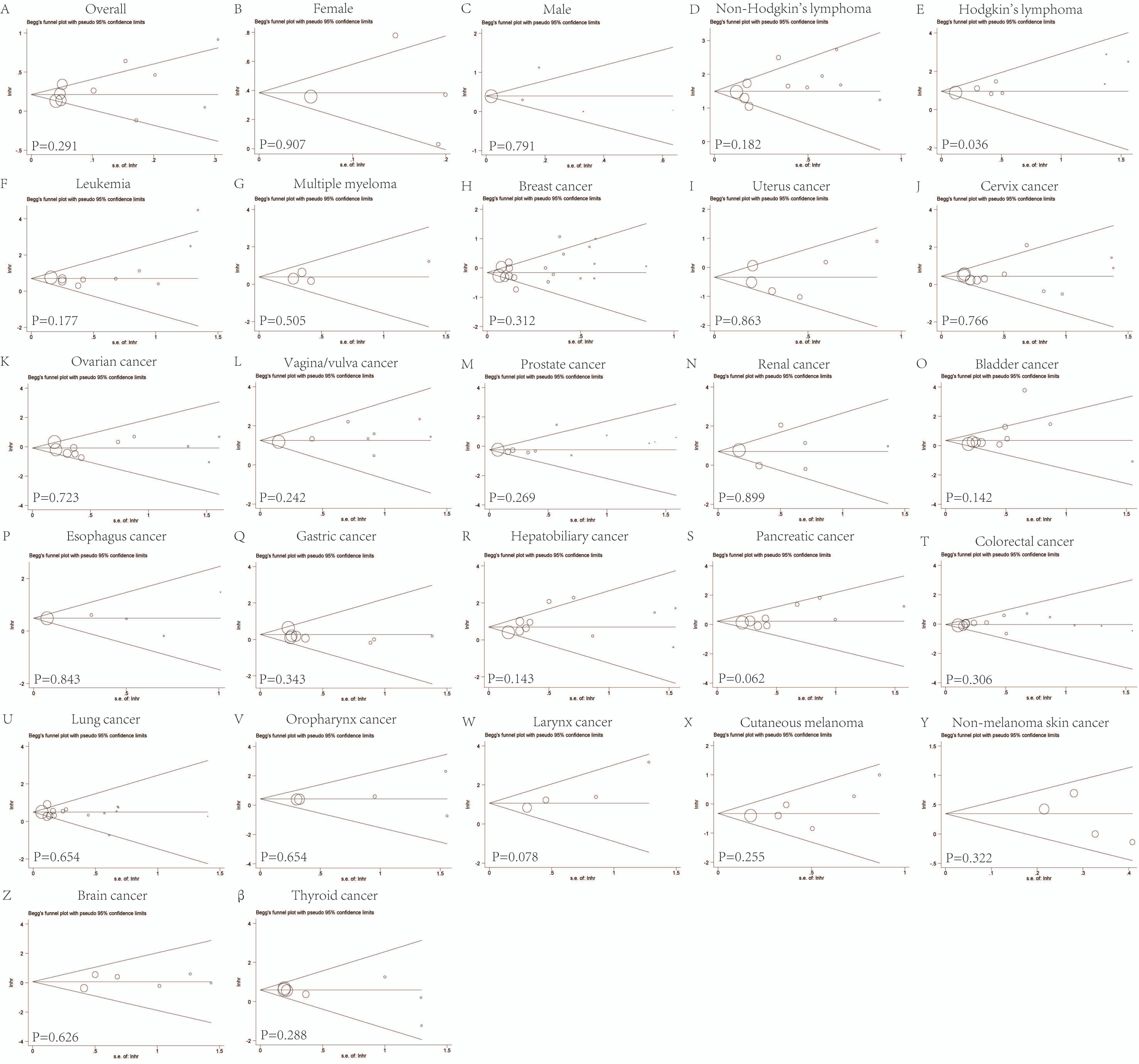

Supplement: Supplementary file 4 — Figure S3. Begg’s funnel plots of the publication bias; (A) The overall cancer; (B) The female group; (C) The male group; (D) Non-Hodgkin's lymphoma; (E) Hodgkin's lymphoma; (F) Leukemia; (G) Multiple myeloma; (H) Breast cancer; (I) Uterus cancer; (J) Cervix cancer; (K) Ovarian cancer; (L) Vagina/vulva cancer; (M) Prostate cancer; (N) Renal cancer; (O) Bladder cancer; (P) Esophagus cancer; (Q) Gastric cancer; (R) Hepatobiliary cancer; (S) Pancreatic cancer; (T) Colorectal cancer; (U) Lung cancer; (V) Oropharynx cancer; (W) Larynx cancer; (X) Cutaneous melanoma; (Y) Non-melanoma skin cancer; (Z) Brain cancer; (β) Thyroid cancer. (PDF 1860 kb) [file 13075_2018_1760_MOESM4_ESM.pdf]
