# Supplementary material for: The risks of cancer development in systemic lupus erythematosus (SLE) patients: a systematic review and meta-analysis
Source: Arthritis Res Ther. 2018 Dec 6;20:270. doi: 10.1186/s13075-018-1760-3 (PMC6282326; doi:10.1186/s13075-018-1760-3)
Supplement: Supplementary file 5 — Table S2. Meta-analysis results for included studies diagnosed according to accepted criteria of SLE. (DOCX 2320 kb) [file 13075_2018_1760_MOESM5_ESM.docx]

**Table S2**: Meta-analysis results for included studies diagnosed according to accepted criteria of SLE

| **Variables** | **NO.of studies** | **Effects model** | **SIR (95%CI)** | **I-squared (%)** | **P values** | **Relationship** | **Publication bias** |
| --- | --- | --- | --- | --- | --- | --- | --- |
| **Overall characteristics** | | | | | | |  |
| Overall cancers | 9 | Random | 1.30 (1.15-1.47) | 75.0% | ＜0.001 | Increased risks | None |
| Female | 3 | Fixed | 1.40 (1.26-1.55) | 26.9% | 0.255 | Increased risks | None |
| Male | 4 | Fixed | 1.48 (1.43-1.53) | 0.0% | 0.522 | Increased risks | Existence |
| **SLE associated with Lymphatic and haematopoietic cancers** | | | | | |  |  |
| Non-Hodgkin's lymphoma | 10 | Fixed | 4.81 (4.14-5.59) | 42.0% | 0.078 | Increased risks | None |
| Hodgkin's lymphoma | 5 | Fixed | 2.77 (1.54-4.99) | 0.0% | 0.541 | Increased risks | Existence |
| Leukemia | 8 | Fixed | 1.90 (1.36-2.65) | 39.9% | 0.113 | Increased risks | None |
| Multiple myeloma | 2 | Fixed | 1.95 (1.01-3.74) | 0.0% | 0.676 | Increased risks | None |
| **SLE associated with Reproductive cancers** | | | | | | | |
| Breast cancer | 13 | Random | 0.84 (0.67-1.06) | 64.2% | 0.001 | No association | None |
| Uterus cancer | 4 | Random | 0.64 (0.32-1.31) | 50.5% | 0.109 | No association | None |
| Cervix cancer | 9 | Fixed | 1.55 (1.22-1.96) | 21.3% | 0.254 | Increased risks | None |
| Ovarian cancer | 9 | Fixed | 1.02 (0.79-1.32) | 3.7% | 0.404 | No association | None |
| Vagina/vulva cancer | 7 | Fixed | 4.34(2.50-7.53) | 0.0% | 0.819 | Increased risks | None |
| **SLE associated with Urinary cancers** | | | | | | | |
| Prostate cancer | 9 | Fixed | 0.80 (0.70-0.92) | 26.5% | 0.208 | Decreased risks | None |
| Renal cancer | 4 | Random | 2.90 (0.97–8.70) | 56.3% | 0.076 | No association | None |
| Bladder cancer | 8 | Random | 2.33 (1.13-4.83) | 78.7% | <0.001 | Increased risks | None |
| **SLE associated with Digestive cancers** | | | | | | | |
| Esophagus cancer | 3 | Fixed | 1.63 (1.41-1.87) | 0.0% | 0.389 | Increased risks | None |
| Gastric cancer | 6 | Fixed | 1.42 (1.05-1.92) | 0.0% | 0.686 | Increased risks | None |
| Hepatobiliary cancer | 8 | Random | 2.85 (1.65-4.92) | 60.7% | 0.013 | Increased risks | None |
| Pancreatic cancer | 7 | Fixed | 1.34 (0.91-1.98) | 26.6% | 0.226 | No association | None |
| Colorectal cancer | 11 | Fixed | 0.99 (0.84-1.17) | 0.0% | 0.850 | No association | None |
| **SLE associated with Respiratory cancers** | | | | | | | |
| Lung cancer | 12 | Fixed | 1.40 (1.23-1.59) | 0.0% | 0.833 | Increased risks | None |
| Oropharynx cancer | 4 | Fixed | 1.54 (0.89-2.65) | 0.0% | 0.557 | No association | None |
| Larynx cancer | 3 | Fixed | 2.72 (1.58-4.70) | 40.5% | 0.187 | Increased risks | None |
| **SLE associated with Other cancers** | | | | | | | |
| Cutaneous melanoma | 4 | Fixed | 0.89 (0.58-1.38) | 0.0% | 0.431 | No association | None |
| Non-melanoma skin cancer | 3 | Fixed | 1.33 (0.92-1.92) | 49.5% | 0.138 | No association | None |
| Brain cancer | 5 | Fixed | 1.48 (0.75-2.93) | 0.0% | 0.965 | No association | None |
| Thyroid cancer | 5 | Fixed | 1.82 (1.41–2.36) | 0.0% | 0.893 | Increased risks | None |

SIR: standardized incidence rate; CI: confidence interval;
